# Molecular Stiffening by Macrocycle Clustering

**DOI:** 10.1002/anie.202420880

**Published:** 2025-04-04

**Authors:** Hang Yin, Qian Cheng, Roselyne Rosas, Stéphane Viel, Valerie Monnier, Laurence Charles, Didier Siri, Didier Gigmes, Mehdi Yemloul, Ruibing Wang, Anthony Kermagoret, David Bardelang

**Affiliations:** ^1^ State Key Laboratory of Quality Research in Chinese Medicine Institute of Chinese Medical Sciences University of Macau Taipa Macau China; ^2^ Aix Marseille Univ CNRS, Centrale Marseille FSCM, Spectropole Marseille France; ^3^ Aix Marseille Univ CNRS ICR AMUtech Marseille France; ^4^ Institut Universitaire de France Paris F‐75005 Paris France; ^5^ Aix Marseille Univ CNRS ISM2 AMUtech Marseille France; ^6^ Aix Marseille Univ CNRS CINaM AMUtech Marseille France

**Keywords:** Cucurbituril, Macrocycles, Rigidification, Supramolecular, Tetratopic

## Abstract

Allosteric stiffening of a portion of a protein surface is a strategy used in nature to regulate protein oligomerization and provide crucial functions for cells. However, a similar strategy to selectively control part of a compound dynamics remains elusive. Here we show that cucurbit[*n*]uril (CB[*n*]) macrocycles can bind almost all portions of a tetratopic guest molecule, stiffening the different parts of the guest to different extents. “Host–guest” interactions were found to be instrumental in selectively “freezing” guest molecular motions. The combination of ^1^H‐NMR (1D, 2D), DOSY, VT‐NMR, isothermal titration calorimetry (ITC), mass spectrometry and molecular modelling enabled to highlight the crucial role of cucurbit[8]uril (CB[8]) binding in the selective hardening of relevant portions of the guest molecule. Beyond implications for bioinspired systems mimicking control of a system dynamic to create a new function, this approach has relevance for improving room temperature phosphorescence, and could also be used to allosterically control organocatalysis in water.

## Graphical abstract

Macrocyclic hosts can remotely rigidify part of a molecule, implying that this part need not be included inside a macrocycle to be stiffened. Here, we show that multiple binding by synthetic hosts CB[7] and CB[8] can selectively rigidify fragments of a tetratopic molecule in water causing a gradual stiffening from the periphery to the core. This suggests that protein dynamics (and so function) could be tuned by targeted multiple external binding.

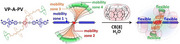

## Introduction

Rigidification is of tremendous importance for several biological functions as for the catalytic activity of thermophilic enzymes,^[^ ^1^ ^]^ in ribosomal translocation,^[^ ^2^ ^]^ or to precisely mark centromere location during cell division.^[^ ^3^ ^]^ On the other hand, the rigidification of protein–protein interfaces by ligand binding has revealed to be a successful approach for function blockage such as the current strategies to lock the rotor movements of ATP synthases and inhibit ATP synthesis in bacteria like mycobacterium tuberculosis, currently a pathogen of concern for the world health organization.^[^ ^4^, ^5^ ^]^ In parallel, protein rigidification has been widely studied in the context of catalysis and its translation in organic solvents.^[^ ^6^, ^7^ ^]^ These examples showcase the importance of mastering not only the structure of molecules but also their dynamics. In supramolecular chemistry, the effects of rigidification upon binding are periodically described,^[^ ^8^, ^9^, ^10^, ^11^, ^12^, ^13^, ^14^ ^]^ and have started to be correlated to desired functions like for improved MRI agents,^[^ ^15^ ^]^ better protein inhibitors,^[^ ^16^ ^]^ room‐temperature phosphorescence in the NIR,^[^ ^17^ ^]^ or for molecular replication.^[^ ^18^ ^]^ Beside covalent modifications, two important methods to reach molecular rigidification are i) the use of metal ions for example to prepare metal–ligand helicates,^[^ ^19^ ^]^ cages,^[^ ^20^ ^]^ or polyhedra,^[^ ^21^ ^]^ and ii) molecular association to restrict conformational freedom (for example for imaging).^[^ ^22^, ^23^, ^24^, ^25^ ^]^ However, and to the best of our knowledge, molecular rigidification by multiple host binding has been seldom reported.^[^ ^26^, ^27^ ^]^

**Figure 1:**
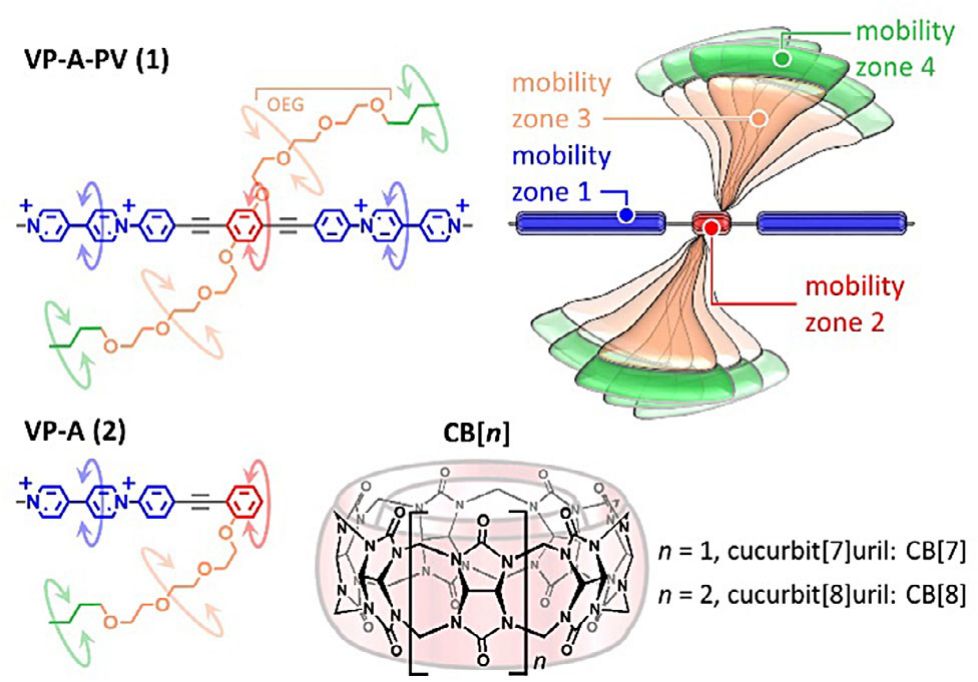
Structures of guest molecules and macrocyclic hosts used in this work (V: viologen, P: phenylene, A: aromatic group).

For some time, we have been working on cucurbit[*n*]urils (CB[*n*], Figure 1),^[^ ^28^, ^29^, ^30^, ^31^ ^]^ rather rigid macrocycles defined by a hydrophobic cavity and two carbonyl laced portals.^[^ ^32^, ^33^, ^34^, ^35^, ^36^, ^37^, ^38^, ^39^, ^40^, ^41^, ^42^, ^43^, ^44^ ^]^

These hosts are prone to bind cations (especially hydrophobic ones) and we used them in a number of cases including crystal engineering,^[^ ^45^ ^]^ supramolecular triangulation,^[^ ^46^ ^]^ or to accelerate the (non‐directional) rotary movement of a guest molecule.^[^ ^47^ ^]^ Recently, we found that compounds of the **VP‐A‐PV** family (Figure 1) afford host:guest 3:2 complexes with cucurbit[8]uril (CB[8]), a first central host binding two of these rather long guests before two more CB[8] bind this complex in periphery.^[^ ^26^ ^]^ The dynamics of central phenylenes was affected by their inclusion in CB[8], the peripheral phenylenes remaining mobile. However, during this work, we repeatedly observed by ITC an unusual break in the titration curve that we could not explain (Figure S1). Intrigued by this result, we repeated the ^1^H NMR titrations and recorded NMR spectra with large excesses of host, and we found a new complex for **VP‐A‐PV** carrying two OEG chains terminated by a butyl group (**VP‐A‐PV** (**1**), see Figures 1 and S2). Here, we show that peripheral CB[8] binding can remotely and selectively restrict the mobility of central parts of a tetratopic guest molecule, so without necessity for these parts to be included in a macrocycle. This guest:host 1:4 complex (**1**•CB[8]_4_) is featured by unusual dynamical behaviour and so we prepared a truncated molecule as a control (**VP‐A** (**2**), Figure 1) to better understand the results obtained with **VP‐A‐PV**.

## Results and Discussion

**Figure 2:**
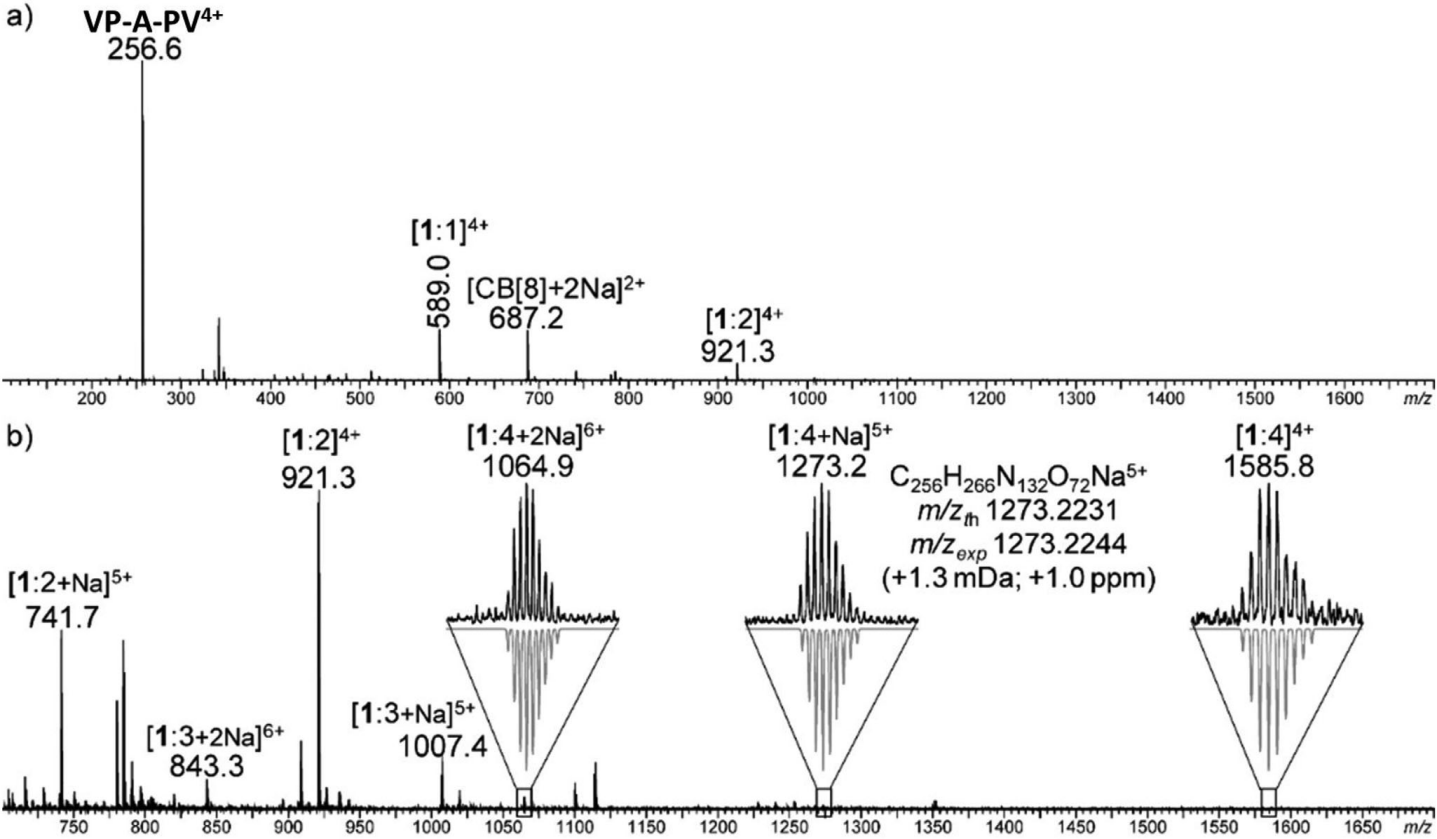
a) Full range and b) expanded 700–1700 *m*/*z* range of the ESI mass spectrum recorded for the **VP‐A‐PV**/CB[8]_4_ mixture after ion separation in the mobility cell of the instrument to enhance the dynamic range of detection and hence allow the weak signals of the targeted 1:4 complex (emphasized in b) to be best detected in spite of the highly abundant individual species (shown in a). Three ionic forms of the 1:4 complex were observed depending of the number of adducted Na^+^, as supported by their isotopic patterns (in black) matching theoretical ones (upside down, in gray) and accurate mass data provided for the *m/z* 1273.2 species in inset.

Syntheses and stoichiometry of CB[8] complexes. The preparation of **VP‐A‐PV** has already been reported.^[^ ^26^ ^]^ **VP‐A** was prepared by successive couplings of relevant precursors based on Sonogashira and Zincke chemistry starting from block **A** (see supporting information and Figures S3–S5). Mass spectrometry of a solution containing **VP‐A‐PV** and excess CB[8] showed the presence of several peaks corresponding to guest:host 1:1, 1:2, 1:3, and 1:4 complexes (Figure 2).

**Figure 3:**
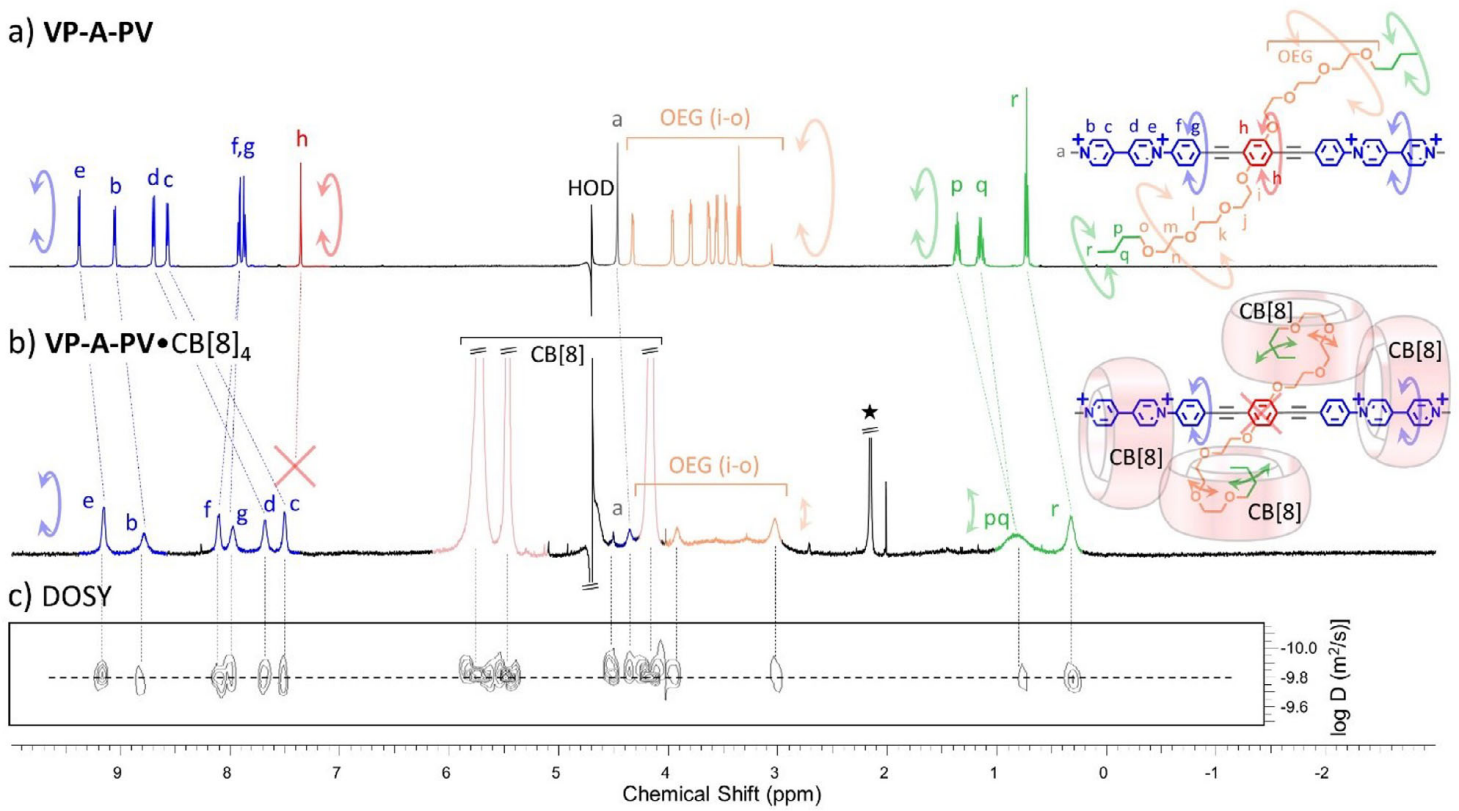
^1^H NMR spectra (500 MHz, D_2_O) of a) **VP‐A‐PV**, b) the **VP‐A‐PV**•CB[8]_4_ complex (300 K), and c) the corresponding Diffusion‐Ordered SpectroscopY (DOSY) spectrum. Note the curved double arrows tentatively accounting for the qualitative molecular motion associated to the colored fragments.

In the tetratopic guest molecule **1**, four recognition sites for CB[8] are present: two viologen groups and two butyl groups. However, they were not expected to be recognized similarly. Indeed, viologens are frequently observed to be included in CB[7] or CB[8]. Butyl groups may also be included but less tightly, especially in our case where they are positioned at the end of a polar OEG chain, known not to be a good fragment for CB recognition. Hence in mass spectra, peaks corresponding to 1:1 and 1:2 CB[8] complexes are easily observed thanks to the good affinity of viologen groups for CB[8] (typically in the order of *K*_a_ = 10^5^–10^6^ M^−1^).^[^ ^35^ ^]^ Conversely, guest:host 1:3 and 1:4 complexes were more difficult to detect presumably due to the lower affinity of CB[8] for OEG‐butyl fragments. Stepping back, supramolecular complexes of weak affinity are known to dissociate in the typical conditions of ESI‐mass spectrometry. Nevertheless, the detection of three distinct isotopic patterns (complex and associated anions) corresponding to the 1:4 stoichiometry allowed us to confirm the existence of a 1:4 guest:host complex both by excellent match with theoretical patterns and by accurate mass measurements. Hence, the presence of high‐stoichiometry complexes, especially the 1:4 complex, is consistent with the presence of a large excess of host and could explain the break observed in ITC measurements, the reorganization from guest:host 2:3 complexes to 1:4 complexes (Figure S2) being energetically demanding.^[^ ^26^ ^]^ Finally, we recorded a ^1^H NMR spectrum of **VP‐A‐PV** in D_2_O with 4 equiv. of CB[8] at 500 MHz and characterized a **1**•CB[8]_4_ complex by this technique (diagnostic peaks for chemical shifts of guest fragments and ratio of integrals consistent with a 1:4 complex, Figures 3 and S6 and S7). With the truncated guest molecule **VP‐A**, the expected 1:2 guest:host complex **2**•CB[8]_2_ was observed (Figures S8–S10 including the right ratio of integrals for signals of host and guest).

**Figure 4:**
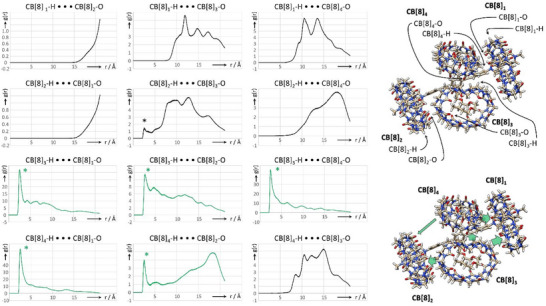
Radial Distribution Functions (RDF) corresponding to distances in the **VP‐A‐PV**•CB[8]_4_ complex (H_2_O, 300 K) between hydrogen atoms of one CB[8] (equatorial H atoms) toward oxygen atoms of one CB[8] rim of the three other CB[8] (the rims considered are the closest to H atoms, those susceptible to engage in C─H•••O hydrogen bonds (abscissas correspond to distances (r) in Å, and ordinates correspond to the *g*(r) function, structures are from the MD trajectory and representative of the complex and green arrows qualitatively illustrate to magnitude of CB[8]–CB[8] interactions between the relevant cucurbiturils.

^1^H‐NMR and modeling of the **VP‐A‐PV**•CB[8]_4_ complex. Surprisingly, the addition of 4 equiv. of CB[8] to a solution of **VP‐A‐PV** in D_2_O at 300 K showed total disappearance of the protons “h” resonance of the central phenylene (Figures 3a,b and S7). Both sets of signals corresponding to the two viologens (“c, d” proton signals) and the two alkyl groups (“p, q, r” proton signals) shifted upfield, in line with CB[8] binding.^[^ ^26^ ^]^ No signal corresponding to free viologen or free butyl was observed, in agreement with a 1:4 guest:host complex. As mentioned before, integrals of ^1^H NMR signals from the guest and the host are in line with a 1:4 stoichiometry (Figure S6) and DOSY confirmed the occurrence of a single complex (featuring signals of the host and the guest) diffusing at a substantially lower speed than the guest or host alone (Figure 3c). All the ^1^H NMR signals corresponding to the **VP‐A‐PV**•CB[8]_4_ complex were broad but signal broadening was different for each fragment of the guest. We first envisioned declined motion due to the molecular weight of the complex (6340 Da) approaching that of some proteins (i.e., for insulin: 5808 Da), but changes in the intra‐complex dynamics appeared more likely, as reflected by the disappearance of the resonance of protons “h”, broadening of resonances corresponding to the OEG chains and more generally heterogeneous signals broadening (Figure 3b). However, raising the temperature above 310 K enabled to see again sharp signals at 500 MHz (Figure S12) including that of protons “h” above 330 K, superimposed to that of protons “c”. In the event, the four binding sites remained complexed by the four CB[8] as suggested by comparing the ^1^H NMR spectra of **1** and **1**•CB[8]_4_ at 330 K (Figure S12). Similar experiments repeated at 800 MHz showed the same trend (Figure S13). They indicated that the central part of **VP‐A‐PV** was locked with respect to the NMR timescale. They also showed that the OEG chains were slightly more mobile (but less than the butyl ends), and that the “narrowest” signals were those corresponding to the aromatic groups (Figure 3, in blue).^[^ ^48^, ^49^ ^]^ Decreasing the temperature to 278 K did not allow us to see clear evidence of coalescence, regardless of the spectrometer frequency (500 or 800 MHz). Importantly, the fact that butyl groups are not included in CB[8] for the side‐chain alone (see control with HO‐(EG)_3_‐Bu, Figure S14) showed that the structure of **VP‐A‐PV** is unique in enabling two more CB[8] to bind the two, otherwise unreachable, butyl groups. This suggests that some interactions between the two types of bound CB[8] are present in the 1:4 complex, stabilizing the two additional CB[8] on butyl stations. We have previously shown how CB[*n*]–CB[*n*] interactions (multiple CH•••O hydrogen bonds between the outside surface of a host and the carbonyl rims of another host)^[^ ^28^ ^]^ played a crucial role in homotropic allosteric binding in water,^[^ ^29^ ^]^ or in the triangular regulation of CB[8] 1:1 complexes.^[^ ^46^ ^]^ Recording 2D ROESY NMR spectra at different mixing times at 800 MHz did not allow us to bring to light these “host–host” interactions, perhaps due to their timescale remaining elusive for NMR. We thus performed molecular dynamics (MD) of the **1**•CB[8]_4_ complex in water to evaluate the stability of this complex and characterize the expected “host–host” interactions. Our analyses revealed that the complex is stable over 100 ns in water at 300 K, meaning that no CB[8] has uncomplexed from its binding site during the simulations (Figure 4, Figure S15 and Supplementary Video).

**Figure 5:**
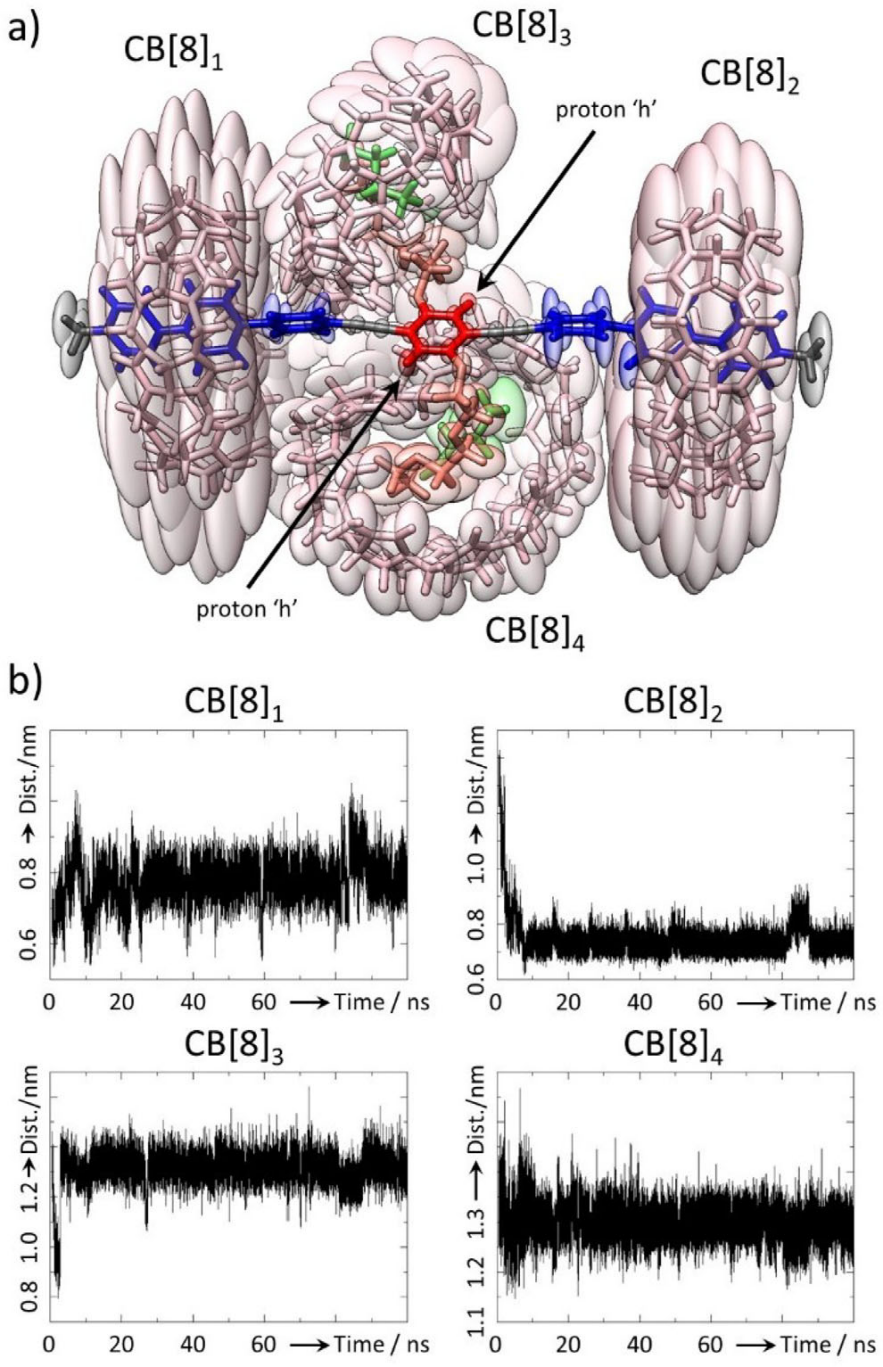
a) Ellipsoid representation of the **VP‐A‐PV**•CB[8]_4_ complex (H_2_O, 300 K) accounting for local positional variabilities around mean atom positions as deduced from molecular dynamics (Chimera, scale factor = 1, smoothing level = 3). Note the relatively large volumes around atoms of CB[8] positioned on viologen stations reflecting positional variations, large volumes also for the side chains included in the two corresponding CB[8], and small volumes for atoms close to the center of the guest with quasi‐minimal volumes for “h” protons indicating that these protons experience minimal displacement during the 100 ns trajectory at 300 K. b) Distances between the barycenter of the guest and that of each CB[8]. While CB[8]_3_ and CB[8]_4_ stay on the viologens at ∼13 Å from the center of **VP‐A‐PV**, CB[8]_1_ and CB[8]_2_ stay complexed at ∼8 Å from the center of the guest.

As expected, the two CB[8] on the main axis stayed complexed on the viologens but are allowed to rotationally move around viologens in a plane nearly perpendicular to the rigid axle (Figure 5).

This is due to the size of CB[8] known to have enough room in its cavity to accommodate both a viologen and a co‐guest at the same time.^[^ ^30^, ^37^, ^50^ ^]^ In the absence of a co‐guest, CB[8] has more freedom to rotate around viologens, and possibly adapt its position to maximize “host–host” interactions. The other two CB[8] stayed complexed on the butyl groups but were much more mobile owing to the flexibility of the guest pendant chains (Figure 5). Nevertheless, they remained confined in a small space and seem “oscillating” between the two peripheral CB[8] “locked” on viologen stations. These oscillations could explain why, despite probably existing on a small timescale, CH•••O interactions were not detected by NMR.

We next searched for statistical data that would allow us to characterize the surmised CH•••O interactions between the CB[8] (CB[8]–CB[8] interactions) to see whether these interactions, ubiquitously observed in the solid state,^[^ ^28^, ^51^ ^]^ and rarely in water,^[^ ^29^ ^]^ were present in this complex. Inter‐host interactions were explored by plotting the radial distribution function (RDF) between the “equatorial” H atoms (plane containing all the methine groups) of the four CB[8] molecules and the 8 oxygen atoms of the closest carbonyl rims of neighboring CB[8] (susceptible to be involved in CH•••O interactions, Figure 4). This parameter accounts for the frequency at which one atom type (H atom of CB[8]) is at a given distance (*x*‐axes of plots of Figure 4) of another atom type (O atom of CB[8]). Several RDF point to CB[8]–CB[8] CH•••O interactions^[^ ^28^, ^51^, ^52^, ^53^ ^]^ with a peak corresponding to distances below the usually considered 2.9 Å limit (sum of H atom and O atom van der Waals radii).^[^ ^54^ ^]^ One “central” cucurbituril CB[8]_3_ is in interaction with CB[8]_1_, CB[8]_2_, and CB[8]_4_ (Figure 4) while the other “central” one, CB[8]_4_, interacts with CB[8]_1_ and CB[8]_2_. These results support experimental data about CB[8]–CB[8] interactions in the **VP‐A‐PV**•CB[8]_4_ complex.

**Figure 6:**
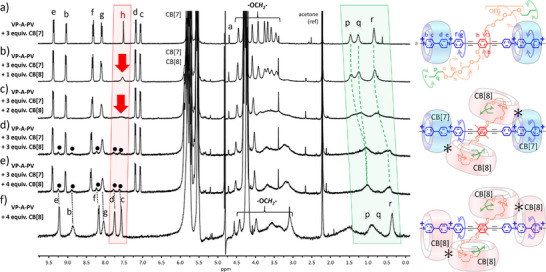
^1^H NMR spectra (500 MHz, D_2_O) of a) the **VP‐A‐PV**•CB[7]_2_ complex (300 K) before addition of CB[8], b), c), d), e) forming a new **VP‐A‐PV**•CB[7]_2_•CB8]_2_ complex (300 K). Note the gradual disappearance of proton “h” signal and the upfield shift of signals of protons corresponding to butyl groups (acetone: reference). Excess CB[7] was used to ensure quasi‐quantitative binding of 2 CB[7] on the 2 viologens and did not impact CB[8] binding as the additional CB[7] could not interact with the guest side‐arms. (•: signals corresponding to a small amount of the **VP‐A‐PV**•CB[8]_4_ complex as shown in (f), ✱ is for the proposed “host–host” interactions)).

We next used **VP‐A** as a control. The addition of 2 equiv. of CB[8] to an aqueous solution of this guest led to CB[8] binding both on the viologen^[^ ^30^, ^55^ ^]^ and the butyl moieties (upfield shifts of “c, d”, and “s, t, u”, proton signals, Figures S8–S10). As the butyl part of monobutyl‐tri(ethylene glycol) is not included in CB[8] in D_2_O (Figure S14), the CB[8]•viologen part plays an important role in stabilizing the CB[8]•butyl binding, most likely by CB[8]–CB[8] interactions. As for **VP‐A‐PV**, the ^1^H NMR spectrum at 300 K of **VP‐A**•CB[8]_2_ (ratio of guest:host integrals ≈ 1:2) showed very broad “h, i, j, k” proton signals of the phenylene consistent with a rigidification of block **A**. ^1^H NMR spectra of **VP‐A**•CB[8]_2_ at low temperature (Figure S16) showed complete disappearance of these signals. However, above 305 K resonances corresponding to phenylene protons were clearly observed, owing to some retrieved conformational freedom for block **A**. Conversely, signals corresponding to the butyl group were broad at 300 K, reflecting a relative residual flexibility while complexed in CB[8], and they became almost undistinguishable from noise at low temperature (in line with frozen motion of block **A**). As for **VP‐A‐PV**, no coalescence could be observed for the **VP‐A**•CB[8]_2_ complex. These results prompted us to experimentally quantify signal broadening of the different guest fragments, but signal overlapping of protons h resonance with those of protons f and g hampered further work in this direction (Figures S12 and S13). However, the use of CB[7] proved useful in affording a slightly different ^1^H NMR for a similar, 1:4 guest:host complex (Figure 6).

“Hybrid” **VP‐A‐PV**•CB[7]_2_•CB[8]_2_ complex. Preliminary tests featuring a mixture of CB[7] and CB[8] in **VP‐A** D_2_O solutions showed a clear preference of the viologen part for CB[7]. With this host alone, single binding by CB[7] on the viologen unit^[^ ^31^, ^56^, ^57^ ^]^ of **VP‐A** was observed by ^1^H NMR (Figure S17), and confirmed by ITC (*K*_a_ = 2.05 (± 0.30) × 10^6^ M^−1^, Figure S18). When CB[8] was added to a solution of the **VP‐A**•CB[7] complex, the signals corresponding to the butyl group started to become broad and to shift to higher field (Figure S19). With this in mind, we then focused on the larger guest. When two or more equivalents of CB[7] were added to a D_2_O solution of **VP‐A‐PV**, a 1:2 complex formed with two CB[7] on the two viologens (Figures S20 and S21). Indeed, signals of the upfield shifted protons “c” and “d” were diagnostic of the presence of CB[7] on viologen stations,^[^ ^31^, ^56^, ^57^ ^]^ and were distinct from those corresponding to CB[8] on viologens (Figure 6). As for **VP‐A**, more CB[7] did not allow us to complex the butyl groups of **VP‐A‐PV**. These results were confirmed by ITC (Figure S22, *K*_a_ = 3.15 ± 0.42 × 10^5^ M^−1^ for each site, a value that is slightly lower than that for **VP‐A** with CB[7]). However, when CB[8] was added to this mixture (Figure 6 and Figures S23–S25), a tremendous effect was observed as the signal of protons “h” disappeared (after addition of 2 equiv. of CB[8]) and all resonances corresponding to the side‐arms became broad, in line with a form of central rigidification. Noticeably, at 1 equiv. of host, the intensity of the resonance due to protons h dramatically decreased but no splitting was observed, which seemed to exclude the possibility of signal disappearance caused by multiple equilibria (two forms exchanging at an intermediate rate). As there was only 1 CB[8] in the mixture, only one side of the phenylene (one of the two h protons) could be affected. This was supported by variable temperature ^1^H NMR spectra of D_2_O solutions of **VP‐A**•CB[8]_2_ (Figure S16) showing that all signals of the phenylene are decreasing in intensity without any shift in resonances potentially caused by host complexation. Overall, about all guest protons behave as local probes of internal mobility of the complexes. The signals of viologens always remained relatively sharp, thereby reflecting unhindered rotation at the ends of the principal axis of the guest molecules. This last observation also allowed us to exclude the possibility of signal broadening due to an impacted global tumbling upon complexation (molecular weight of 6340 Da for the 1:4 complex) because several resonances remained relatively sharp. Spinning at the magic‐angle a D_2_O solution of the **1**•CB[7]_2_•CB[8]_2_ complex at 4 kHz in the rotor of a solid‐state NMR spectrometer at 400 MHz (Figure S26) did not allow us to recover the missing protons “h” resonance, suggesting that the corresponding signal loss was not due to a very slow tumbling of the complex in water. With 3 equiv. of CB[8], signals of “p, q, r” protons were clearly upfield shifted, in agreement with butyl groups complexed by CB[8]. These results confirmed the crucial role of CB[8] both for butyl chain binding and for **VP‐A‐PV** stiffening. Again, “host–host” (CB[7]–CB[8]) interactions are strongly suspected to play a major role in rigidifying the core of the **1**•CB[7]_2_•CB[8]_2_ complex. On several occasions, we tried to determine the binding constant *K*_a__CB[8]‐Bu_ for the binding of CB[8] toward butyl groups by competition NMR but this was not possible for the **1**•CB[8]_4_ complex since competitors collect some host from this complex leading to complex mixtures containing the previously reported **1**_2_•CB[8]_3_ complex (Figure S27). For the **1**•CB[7]_2_•CB[8]_2_ complex (not suffering this limitation), we had to find a competitor i) binding CB[8] with an affinity similar to that of butyl groups of this complex, for CB[8], and ii) at the same time showing no or limited binding for CB[7]. After having tested several compounds, DiQuat appeared to be a good competitor. Using the reference value of *K*_a CB[8]•DiQuat_ reported by Kaifer (Figure S28) and assuming identical CB[8] binding constants for the two butyl groups of **VP‐A‐PV**•CB[7]_2_•CB[8]_2_,^[^ ^58^ ^]^ we estimated a *K*_a_ value of ≈ 9 × 10^6^ M^−2^ (see supporting information). This result supports again the occurrence of CB–CB interactions since the HO‐(EG)_3_‐Bu molecule is not included by CB[8] in water (Figure S14)!

Coming back to the mixed host titration (Figure 6), a slight excess of CB[8] was necessary to get the maximum amount of the **1**•CB[7]_2_•CB[8]_2_ complex (Figure 6d) and this could be due to weaker CB[7]–CB[8] interactions (fewer CH•••O interactions) compared to CB[8]–CB[8] interactions in the **VP‐A‐PV**•CB[8]_4_ complex. Indeed, with the two butyl‐bound CB[8] hosts navigating in a preorganized way between the two other facing cucurbiturils, the probabilities to maximize multiple CH•••O interactions are higher for two viologen bound CB[8] compared to two viologen bound CB[7]. The excess of CB[8] resulted in the appearance of a new set of signals (labeled with • in Figure 6) assigned to the **VP‐A‐PV**•CB[8]_4_ complex, in competition with the **VP‐A‐PV**•CB[7]_2_•CB[8]_2_ complex. However, in these conditions, the “hybrid” hetero‐host complex is much favored, compared to that of the homo‐host analog representing less than 10% of complexed **VP‐A‐PV**.

**Figure 7:**
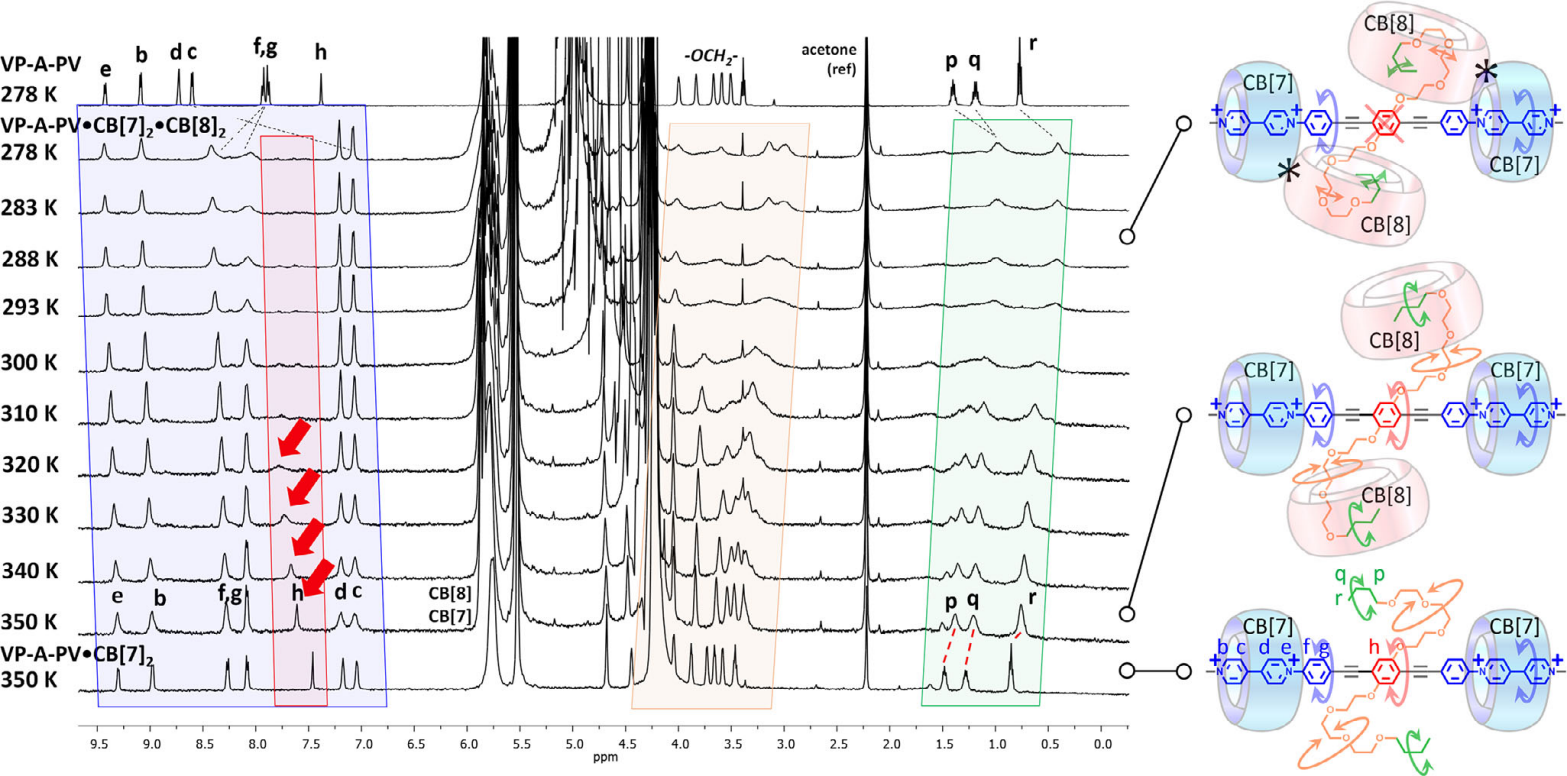
Variable‐temperature ^1^H NMR spectra (500 MHz, D_2_O) of the **VP‐A‐PV**•CB[7]_2_•CB[8]_2_ complex with ^1^H NMR spectra of solutions of **VP‐A‐PV** at 278 K (top) and of **VP‐A‐PV**•CB[7]_2_ at 350 K (bottom) for comparisons.

As for the **VP‐A‐PV**•CB[8]_4_ complex, increasing the temperature of a solution containing the **VP‐A‐PV**•CB[7]_2_•CB[8]_2_ complex enabled the lost resonance at ∼7.65 ppm to reappear, and all resonances became sharp again (Figures 7 and S29).

Rising the temperature of the solution probably enabled to weaken the CB[*n*]–CB[*n*] interactions and to restore some core conformational freedom. As for **VP‐A**, the signal corresponding to block **A** (protons “h”) of **VP‐A‐PV** with CB[7] and CB[8] became very broad below 300 K with disappearance of the resonance again supporting gradual stiffening getting closer to the center of the complex. While temperature increase undoubtedly caused nearly all signals to become relatively sharp again, an important question was whether CB[8] remained complexed at high temperature. As can be seen at the bottom of Figure 7, the signals corresponding to the butyl chains (“p, q, r”) shifted upfield even at 350 K compared to corresponding resonances at the same temperature for the **VP‐A‐PV**•CB[7]_2_ complex supporting that CB[8] remained significantly complexed on the butyl arms in the **VP‐A‐PV**•CB[7]_2_•CB[8]_2_ complex at high temperature. Thus, CB[8] played an important role in remotely rigidifying the complex core at room temperature most likely by CB[8]–CB[7] interactions, but some conformational freedom could be recovered at elevated temperatures.

Relaxation times, host exchange and effect of chemical competition. With signals in the aromatic region better resolved for the mixed host complex, we decided to measure relaxation times *T*_1_ and *T*_2_ to determine the magnitude of signal broadening as a function of temperature. Values for relevant protons are shown in Table 1. From these data, it appeared that *T*_2_ values of protons “b”, “e”, “f” and “g” remained the same order of magnitude (between each other and with temperature), but *T*_2_ values for protons “h” remained about five times smaller compared to *T*_2_ values of other protons (whatever temperature), which was in line with a more rapid relaxation around the central phenylene probably caused by slower cycle reorientations. Despite experiencing a similar environment, values of *T*_2_ for “g” and “h” protons, were different (by factors comprised between ∼1.4 and ∼8.2 depending on temperature) pointing to a central stiffening of the **VP‐A‐PV**•CB[7]_2_•CB[8]_2_ complex. Comparisons at given temperatures between other signals from other protons show that *T*_2_ values remain very similar.

**Table 1:** Longitudinal (*T*_1_) and transverse (*T*_2_) relaxation times of selected protons of the **VP‐A‐PV**•CB[7]_2_•CB[8]_2_ complex as a function of temperature in D_2_O.^a)^

	Proton “b”		Proton “e”		Proton “f”		Proton “g”		Proton “h”	
*T/K*	*T*_1_/ms	*T*_2_/ms	*T*_1_/ms	*T*_2_/ms	*T*_1_/ms	*T*_2_/ms	*T*_1_/ms	*T*_2_/ms	*T*_1_/ms	*T*_2_/ms^b)^
360	4.72	0.56	3.23	0.47	4.45	0.54	6.40	0.57	2.53	0.09
355	4.80	0.48	3.91	0.27	5.03	0.48	6.45	0.56	2.40	0.10
345	5.66	0.70	3.20	0.47	4.09	0.48	6.14	0.41	2.30	0.05
340	4.90	0.52	3.18	0.51	2.89	0.42	4.53	0.30	1.85	0.06
335	4.30	0.55	2.95	0.52	3.38	0.41	4.72	0.25	1.94	0.17

^a)^ For proton labeling, see Figure 7.

^b)^ The spin‐spin relaxation time *T*_2_ corresponding to proton “h” is small compared to identical values for other protons of the complex.

This central stiffening is reminiscent of gels or micelles formation for which some parts of proton resonances are absent from ^1^H NMR spectra due to intense rigidification compared to pendant arms remaining sufficiently mobile to be featured by characteristic NMR signals.^[^ ^59^, ^60^, ^61^ ^]^

**Figure 8:**
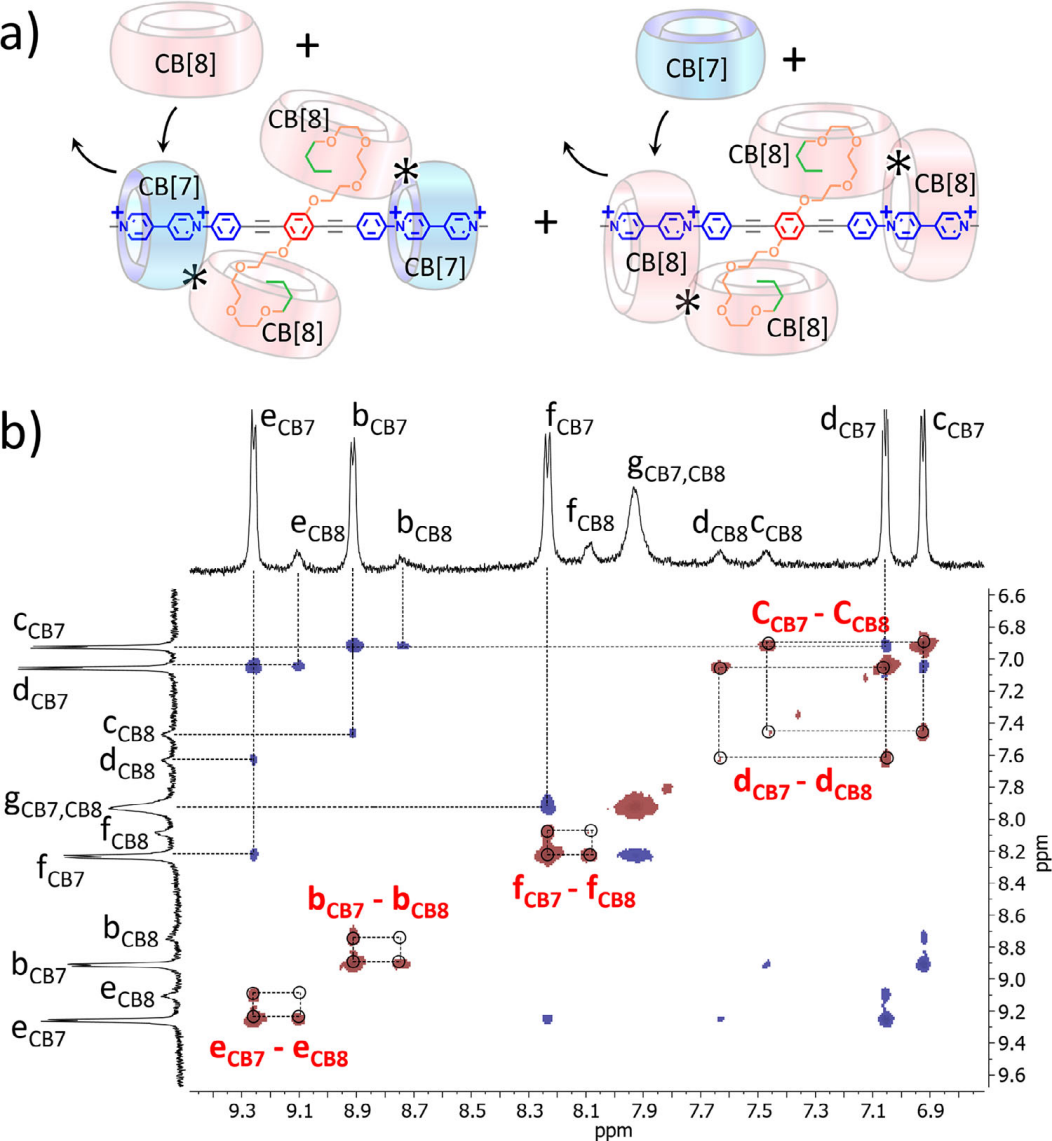
a) Proposed CB[7]‐CB[8] exchange on viologen stations of the two complexes **VP‐A‐PV**•CB[7]_2_•CB[8]_2_ and **VP‐A‐PV**•CB[8]_4_ and b) part of the 500 MHz ROESY spectrum of a solution of **VP‐A‐PV** with 3 equiv. CB[7] and 3 equiv. CB[8] in D_2_O (mixing time 400 ms).

As mentioned before, ROESY spectra recorded at different mixing times and different frequencies did not allow to characterize CB[*n*]–CB[*n*] interactions, but unexpected cross‐correlations were observed in the aromatic region (Figures 8 and S30–S32). To be sure to saturate the viologen binding sites with CB[7] and because this host doesn't bind the butyl groups, we used 3 equiv. of CB[7]. As can be seen in Figure 6, 3 equiv. of CB[8] were required to form the final complex **VP‐A‐PV**•CB[7]_2_•CB[8]_2_ but, in these conditions, about 10% of the **VP‐A‐PV**•CB[8]_4_ complex were observed, conditions presenting excess CB[7] and CB[8] in the mixture. In this case, exchange cross‐correlations (EXSY)^[^ ^62^, ^63^ ^]^ could be observed at different mixing times (Figures 8b and S30–S32) supporting exchange between CB[7] and CB[8] on the viologens of **VP‐A‐PV** and so some dynamics of host exchange in D_2_O solution at 300 K.

Indeed, beside the expected cross‐correlations (opposite sign compared to diagonal peaks) for NOE effects related to atoms close in space (i.e., bCB7‐cCB7, or dCB7‐eCB7), new peaks were present with the same sign as that of the diagonal indicating an apparent exchange between identical protons but in different environments (CB[7] or CB[8], bCB7‐bCB8, cCB7‐cCB8, dCB7‐dCB8, eCB7‐eCB8, and fCB7‐fCB8). So even in a mixture of **1**•CB[7]_2_•CB[8]_2_ and **1**•CB[8]_4_ complexes, still featured by rigidified complexes at 300 K, the hosts still dynamically exchange, at least on the peripheral viologen stations of **VP‐A‐PV**. These results made us wondering about necessary but sufficient conditions to promote **VP‐A‐PV** rigidification. Starting from the **VP‐A‐PV**•CB[7]_2_ complex, only 1 equiv. of CB[8] is (necessary and) sufficient to make the signal of protons “h” dramatically decrease (see Figure 6b). Then, the second equivalent of CB[8] seems serving as a “dynamic anchor” to further rigidify the **VP‐A‐PV**•CB[7]_2_•CB[8]_2_ complex.

Because CB[7] and CB[8] are respectively sorted on the viologens and the butyl groups, we wondered if a suitable competitor could selectively complex CB[8] (better than the butyl groups of **VP‐A‐PV**) thereby allowing the central phenylene to retrieve some rotational freedom by a chemical stimulus. The competitor 3,5‐dimethylamantadine hydrochloride (DMA) was chosen on account of its high and selective binding for CB[8].^[^ ^64^ ^]^ Pleasingly, the addition of 2 equiv. of DMA to a solution of the **VP‐A‐PV**•CB[7]_2_•CB[8]_2_ complex produced the **VP‐A‐PV**•CB[7]_2_ complex allowing the signal of proton “h” to be totally recovered (Figure S33). This result further demonstrated the role of CB[8] in gradually stiffening the complex as a function of the distance from the center (mobility zones of Figure 1) following the order: 1) viologens and peripheral phenylenes < 2) butyl groups < 3) OEG chains < 4) central phenylene.

## Conclusions

Molecular stiffening is of paramount importance for some biological functions (i.e,. membrane rigidification by cholesterol,^[^ ^65^ ^]^ calcium triggered E‐cadherin stiffening and dimerization,^[^ ^66^ ^]^ or in CRABP2 dimerization involved in specific gene transcription).^[^ ^67^ ^]^ However, little has been done for mastering the dynamics of supramolecular assemblies. In this work, we showed that a linear shaft featuring two viologen stations and flanked by two flexible monobutyl‐tri(ethylene glycol) arms has its different fragments dramatically impacted by CB[*n*] binding and inter‐host CB[*n*]–CB[*n*] interactions. Upon complexation by four cucurbiturils in water and while the viologen fragments keep a relative mobility, the core of the complex dramatically stiffened (central phenylene and pendant OEG chains). CB[*n*]–CB[*n*] interactions (multiple non‐classical C─H•••O hydrogen bonds), often considered negatively since at the origin of the poor solubility of cucurbiturils are here proposed to be pivotal for the seemingly decoupled rigidification of several fragments of the molecular guest **VP‐A‐PV**.^[^ ^28^, ^51^ ^]^ Rising temperature or adding a relevant CB[8] competitor weakened or cancelled the host:host supramolecular interactions and allowed the complex core to recover some conformational freedom. Perhaps the closest field related to CB[*n*]‐induced rigidification is that of fluorescent molecular rotors for which host binding restricts rotational degrees of freedom often resulting in improved emissions^[^ ^23^, ^27^, ^68^, ^69^, ^70^, ^71^ ^]^ especially room‐temperature phosphorescence.^[^ ^72^, ^73^, ^74^, ^75^, ^76^ ^]^ This work broadens the field by showing that i) non‐emissive fragments can also be stiffened, ii), remotely and (iii) CB[*n*]–CB[*n*] interactions can be used purposefully to further improve the rigidification solely provided by CB[*n*] binding. Besides evident implications for molecular rotors, molecular stiffening is also relevant to the domain of proteins. The gradual change in local dynamics from a relatively rigid center (central phenylene) to more flexible peripheral groups (butyls and viologens) is reminiscent of the intrinsic dynamics of some proteins.^[^ ^77^ ^]^ The selective stiffening of domains of proteins caused by CB[*n*] binding could trigger protein oligomerization and so enable to control access to functions of the oligomerized state. Pioneering work about CB[*n*] binding toward peptides and proteins is ongoing.^[^ ^78^, ^79^, ^80^, ^81^, ^82^ ^]^ Surface residues of the following types: benzyl (Phe), 4‐hydroxylbenzyl (Tyr), isobutyl (Leu) or alkylamines (Lys) could be used as anchor groups to aggregate cucurbiturils on a certain part of a protein, macrocycles acting as triggers of surface stiffening to control a protein function. Finally, prediction tools of protein stiffening could be used to determine which protein (and protein domain) can be sensitive to macrocycle binding toward controlling domain rigidification.^[^ ^83^, ^84^ ^]^

## Supporting Information

Preparation of **VP‐A** and **VP‐A‐PV**, of the following cucurbituril complexes and their characterizations can be found in the Supporting Information: **VP‐A**•CB[7], **VP‐A**•CB[8]_2_, **VP‐A‐PV**•CB[7]_2_, **VP‐A‐PV**•CB[8]_4_, and **VP‐A‐PV**•CB[7]_2_•CB[8]_2_. Additional procedures, NMR spectra, ITC thermograms, mass spectra and modelling data can also be found in the Supporting Information. The authors have cited additional references within the Supporting Information.^[^ ^26^, ^28^, ^85^, ^86^, ^87^, ^88^, ^89^ ^]^

## Conflict of Interests

The authors declare no conflict of interest.

## Supporting information



Supporting Information

Supporting Information

## Data Availability

The data that support the findings of this study are available in the supplementary material of this article.
