# Supplementary material for: Molecular Stiffening by Macrocycle Clustering
Source: Angew Chem Int Ed Engl. 2025 Apr 4;64(22):e202420880. doi: 10.1002/anie.202420880 (PMC12105694; doi:10.1002/anie.202420880)
Supplement: Supplementary file 2 — Supporting Information [file ANIE-64-e202420880-s001.docx]

Supporting Information for

**Molecular stiffening by macrocycle clustering**

Hang Yin, Qian Cheng, Roselyne Rosas, Stéphane Viel, Valérie Monnier, Laurence Charles, Didier Siri, Didier Gigmes, Mehdi Yemloul, Ruibing Wang, Anthony Kermagoret and David Bardelang

Table of Contents

- Experimental procedures

1/ Chemical compounds ------------------------------------------------------------------------------------ S2

2/ NMR Measurements ------------------------------------------------------------------------------------- S2

3/ Mass spectrometry --------------------------------------------------------------------------------------- S3

4/ Isothermal Titration Calorimetry ----------------------------------------------------------------------- S3

5/ Molecular modelling -------------------------------------------------------------------------------------- S3

- Additional data

6/ Isothermal Titration Calorimetry (ITC) for **VP-A-PV** with CB[8] in water -------------------- S4

7/ ^1^H NMR titration of **VP-A-PV** with CB[8] ----------------------------------------------------------- S5

8/ Syntheses and characterizations of precursors --------------------------------------------------- S6

9/ Synthesis and characterizations of **VP-A** ----------------------------------------------------------- S6

10/ Preparation and NMR spectra of the **VP-A-PV**•CB[8]_4_ complex ---------------------------- S10

11/ Preparation and NMR spectra of the **VP-A**•CB[8]_2_ complex --------------------------------- S12

12/ Variable temperature NMR spectra of **VP-A-PV** ------------------------------------------------ S14

13/ 500 MHz and 800 MHz VT NMR spectra of the **VP-A-PV**•CB[8]_4_ complex -------------- S15

14/ NMR tests of HO-(EG)_3_-Bu binding by CB[7] and CB[8] -------------------------------------- S16

15/ Superimposed structures from the MD trajectory of the **VP-A-PV**●CB[8]_4_ complex ---- S17

16/ Variable temperature NMR spectra of **VP-A**•CB[8]_2_ -------------------------------------------- S18

17/ Preparation, NMR spectra and ITC of the **VP-A**•CB[7] complex ---------------------------- S19

18/ Preparation, NMR spectra and ITC of the **VP-A-PV**•CB[7]_2_ complex ---------------------- S21

19/ Preparation and NMR spectra of the “hybrid” **VP-A-PV**•CB[7]_2_•CB[8]_2_ complex -------- S23

20/ ^1^H MAS NMR spectrum of **VP-A-PV**•CB[7]_2_•CB[8]_2_ -------------------------------------------- S25

21/ Determination of binding constants by competition NMR-------------------------------------- S26

22/ Variable temperature ^1^H NMR spectra of **VP-A-PV**•CB[7]_2_•CB[8]_2_ ------------------------- S29

23/ ROESY spectra of **VP-A-PV**•CB[7]_2_•CB[8]_2_ ------------------------------------------------------ S29

24/ ^1^H NMR spectra of **VP-A-PV**•CB[7]_2_•CB[8]_2_ with **DMA** as selective CB[8] competitor - S32

25/ References ------------------------------------------------------------------------------------------------ S33

- Experimental procedures

***1/ Chemical compounds.*** Amberlite®IRA 400 chloride form, Amberlite®IRA 900 chloride form, tri(ethylene glycol) monobutyl ether, 4-toluenesulfonyl chloride, NEt_3_, ICH_3_, 2-Iodophenol, Pd(PPh_3_)_4_, CuI, K_2_CO_3_, KPF_6_, ICl, hydroquinone, p-ethynyl-aniline, 3,5-dimethylamantadine hydrochloride (**DMA**), solvents (CH_2_Cl_2_, DMSO-D6, D_2_O, CDCl_3_, CD_3_OD, acetone-D6, acetonitrile, pentane, heptane, EtOAc, MeOH), DiQuat were purchased from Aldrich, Acros or TCI and used without further purification. CB[7] and CB[8] were prepared according to a previous paper.^[1]^ 1-(2,4-Dinitrophenyl)-4-(pyridin-4-yl)pyridinium chloride was prepared according to the literature.^[2]^ **VP-A-PV** was prepared according to a previously reported procedure.^[3]^

***2/* NMR Measurements.** NMR spectra were recorded on BRUKER Avance III nanobay 300 or 400 spectrometers, a BRUKER Avance HD 500 spectrometer and a BRUKER Avance III 800SB spectrometer (^1^H-NMR frequencies 300.13, 400.13, 500.13 and 800.15 MHz and ^13^C-NMR frequencies 75.46, 100.60, and 125.75 MHz respectively) using D_2_O as the solvent and a watergate sequence (water suppress) when necessary (potentially affecting signals and integrals near the signal suppressed). Acetone was also used when necessary as a reference. Splitting patterns are indicated as follows: s, singlet; d, doublet; t, triplet; m, multiplet. More specifically, detailed conditions are listed hereafter as a function of the instrument used to implement the experiments. **300MHz.** All spectra were recorded at 300K on the 300 MHz spectrometer equipped with a multi-nuclei 5mm BBFO probe with Z-gradient. COSY spectra were obtained using a gradient sequence *"cosygpppqf"*, an *F2* spectral width of 13.34 ppm (1K data points), an *F1* spectral width of 256 *t1* increments and 8 scans, before processing with SI2=1K and SI1=1k and pure sine window functions applied to both dimensions. **400MHz.** All spectra were recorded at 300K on the 400 MHz spectrometer equipped with a multi-nuclei 5mm BBFO probe with Z-gradient. The COSY spectra were obtained using a gradient sequence *"cosygpqf"*, an *F2* spectral width of 13.34 ppm (1K data points), an *F1* spectral width of 256 *t1* increments and 16 scans, before processing with SI2=1K and SI1=1k and pure sine window functions applied to both dimensions. The HMQC spectrum was obtained using a gradient sequence *"hmqcgpqf"*, an *F2* spectral width of 10.11 ppm (1K data points), an *F1* spectral width of 165 ppm with 256 *t1* increments, 8 scans and a delay time of 1.5 s. The sequence was optimized for J(^13^C-^1^H) = 145 Hz, and the spectrum processed with SI2=1K and SI1=1k and pure cosine squared sine window functions applied to both dimensions. **500MHz.** All spectra were recorded at 300K unless otherwise noted on the 500MHz spectrometer equipped with a multi-nuclei 5mm BBFO probe with Z-gradient using Bruker Topspin (version 3.6.2). ROESY spectra were obtained using the NMR sequence *"roesyphpr.2"* including water suppression, an *F2* spectral width of 12 ppm (2K data points), an *F1* spectral width of 256 *t1* increments, 32 scans, and processed with SI2=2K and SI1=1k and pure cosine squared sine window functions applied to both dimensions. To evidence the exchange phenomenon, two ROESY spectra were recorded using 200 ms and 400 ms for the mixing time. 2D DOSY experiments were performed using a pulse sequence incorporating bipolar gradient pulses and a longitudinal eddy current delay (LED). A total of 16 gradient values were linearly sampled from 6% to 95%. 16 scans were recorded (32k data points) for a total acquisition time of *ca.* 32 min. The gradient pulse duration (∂/2) and the diffusion time (∆) were set to 2 ms and 80 ms respectively, with a gradient recovery delay of 0.1 ms and a LED of 5 ms. The DOSY spectra were simply obtained using the single-exponential fitting routine of the EDDOSY feature of the TopSpin software from Bruker. **800MHz.** All spectra were recorded at several temperatures on the 800MHz spectrometer equipped with a Triple Resonance ^1^H/^13^C/^15^N 5mm TXI probe with Z-gradient using Bruker Topspin (version 3.5.PL7). ROESY spectra were obtained using the NMR sequence *"roesyadjsphpr"* including water suppression, an *F2* spectral width of 13ppm (4K data points), an *F1* spectral width of 512 *t1* increments, a delay time of 2s and a mixing time of 200 ms for 64 scans, before processing with SI2=2K and SI1=2k and pure cosine squared sine window functions applied to both dimensions. **^1^H MAS NMR.** The ^1^H MAS NMR spectrum was performed on a Bruker Avance III WB 400 spectrometer (Spectropole, Aix-Marseille Univ, France) at a spinning rate of 4 kHz.

***3/* Mass spectrometry.** High resolution mass spectrometry experiments were performed with a Synapt G2 HDMS mass spectrometer (Waters, Manchester, UK) equipped with a pneumatically assisted electrospray ionization (ESI) source operated at 35°C in the positive ion mode (electrospray voltage: +2.8 kV; declustering potential: +20 V; nebulizing gas flow: N_2_, 100 L.h^–1^). Mass spectra were acquired using an orthogonal acceleration time-of-flight (oa-TOF) mass analyzer. To improve detectability of the weak signals assigned to the targeted complex in presence of the strong signals of individual species, all ions generated in the ESI source were separated in the mobility cell of the instrument prior to mass analysis. The sample (0.2 mM in H_2_O) was diluted (1/2, v/v) in a H_2_O/acetonitrile (80:20, v/v) binary mixture and then injected in the ESI source at a 10 μL.min^–1^ flow rate using a syringe pump. Accurate mass measurements were achieved using an external calibration for the oa-TOF mass analyzer. Instrument control, data acquisition and data processing were performed with the MassLynx 4.1 programs provided by Waters.

***4/* Isothermal Titration Calorimetry.** Isothermal titration calorimetry (ITC) was performed on a Malvern MicroCal PEAQ-ITC. Titration of **VP-A-PV** with CB[8]. Stock solutions of 0.05 mM of CB[8] and 0.25 mM of **VP-A-PV** were prepared by using Milli-Q water. 40 μL of the 0.25 mM **VP-A-PV** solution were loaded in the titration syringe while 280 μL of the 0.05 mM CB[8] solution were introduced in the titration cell. 19 drops mode (0.4 μL for the first drop and 2.0 μL per drop for the others 18 drops), a temperature of 25 °C, a stirring speed of 750 rpm, and a reference power of 10.0 μcal/s were set for the titration process. The results were analyzed using the Malvern MicroCal PEAQ-ITC Analysis Software 1.1.0.1262. Titration of **VP-A** with CB[7]. 40 μL of a 1.0 mM aqueous solution of **VP-A** were loaded in the titration syringe while 280 μL of a 0.06 mM aqueous solution of CB[7] were loaded in the titration cell. 19 drops mode (0.4 μL for the first drop and 2.0 μL per drop for the others 18 drops), a temperature of 25 °C, a stirring speed of 750 rpm, and a reference power of 10.0 μcal/s were set for the titration process. The solutions were prepared in Milli-Q water. The results were analyzed using the Malvern MicroCal PEAQ-ITC Analysis Software 1.1.0.1262. Titration of **VP-A-PV** with CB[7]. Stock solutions of 2 mM of CB[7] and 0.1 mM of **VP-A-PV** were prepared by using Milli-Q water. 40 μL of the 2 mM CB[7] solution were loaded in the titration syringe while 280 μL of the 0.1 mM **VP-A-PV** solution were loaded in the titration cell. 19 drops mode (0.4 μL for the first drop and 2.0 μL per drop for the others 18 drops), a temperature of 25 °C, a stirring speed of 750 rpm, and a reference power of 10.0 μcal/s were set for the titration process. The results were analyzed using the Malvern MicroCal PEAQ-ITC Analysis Software 1.1.0.1262.

***5*/ Molecular modelling.** All Molecular Dynamics calculations were performed with Gromacs 2019 software.^[4]^ The compounds were embedded in a cubic box containing TIP3P water molecules. After an energy minimization to avoid close contacts, we relaxed the volume of the simulation box by performing an NPT simulation of 400 ps duration (300 K, 1 atm, time step of 0.25 fs). Once this initial run was achieved, the simulation was then restarted in the NVT ensemble during 100 ns (300 K, time step of 0.25 fs) using GAFF force field. Radial distribution functions (RDF, Figure 4 of the paper) between particles of type A and type B are defined in the following way:

$${RDF}_{AB}\left( r \right)=\frac{\left\langle\rho_{B}\left( r \right) \right\rangle}{\left\langle\rho_{B} \right\rangle_{local}}=\frac{1}{\left\langle\rho_{B} \right\rangle_{local}}\frac{1}{N_{A}}\sum_{i\in A}^{N_{A}} \sum_{j\in B}^{N_{B}} \frac{\delta\left( r_{ij}-r \right)}{4\pi r^{2}}$$

with $\left\langle\rho_{B}\left( r \right) \right\rangle$ the particle density of type B at a distance $r$ around particles A, and $\left\langle\rho_{B} \right\rangle_{local}$ the particle density of type B averaged over all spheres around particle A with radius $r_{max}$ (half of the box length).

- Additional data

***6*/ *Isothermal Titration Calorimetry (ITC) for VP-A-PV with CB[8] in water***


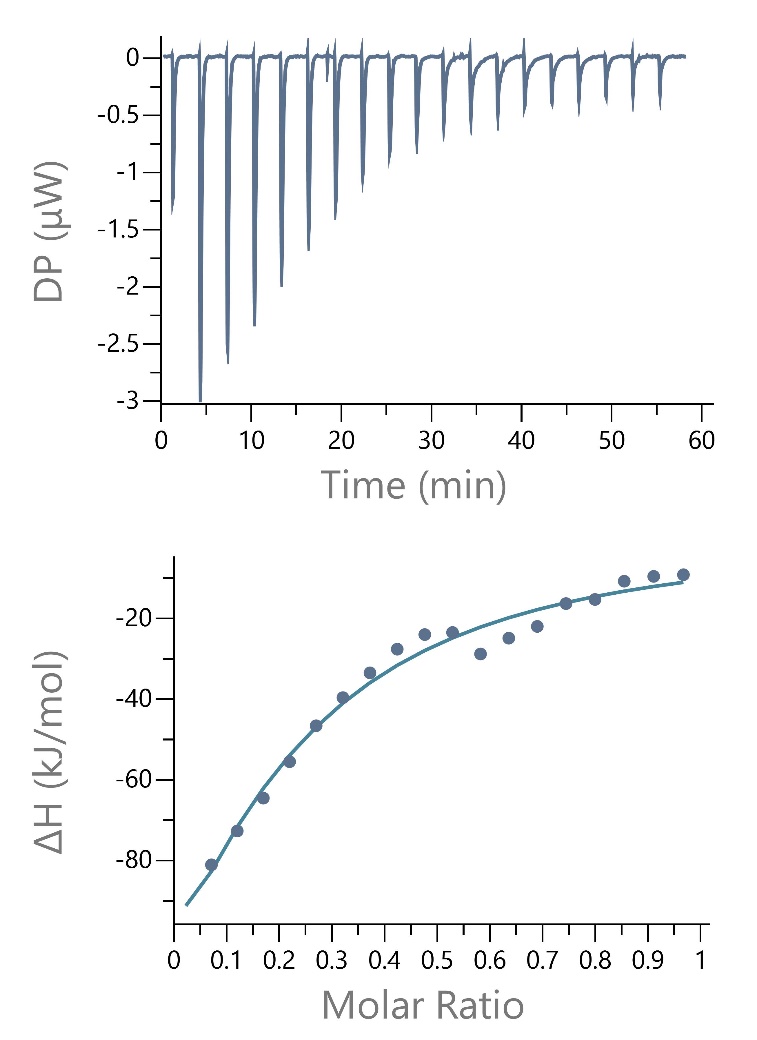


***Figure S1.*** Isothermal titration calorimetry for **VP-A-PV** titrated with CB[8] and showing a break in the curve near the 0.6 molar ratio, close to the 2:3 stoichiometry at which this complex form before evolving toward the 1:4 complex.

***7*/ *^1^H NMR titration of VP-A-PV with CB[8]*.**

***Figure S2.*** ^1^H NMR titration of **VP-A-PV** with CB[8] in D_2_O at 300 K (concentration of CB[8]: 0.5 mM). The sequenced complexation of **VP-A-PV** led to a first complex **VP-A-PV**_2_•CB[8] (with 0.5 equiv of CB[8]), then a second complex **VP-A-PV**_2_•CB[8]_3_ (with 1.8 equiv of CB[8]),^[3]^ and finally to complex **VP-A-PV**•CB[8]_4_ in the presence of 4 equiv. of CB[8].

***8*/ *Syntheses and characterizations of precursors*.**

*Synthesis of 1-​(2,​4-​dinitrophenyl)​-​1'-​methyl-4,​4'-​bipyridinium chloride iodide.*

Compound *1-​(2,​4-​dinitrophenyl)​-​1'-​methyl-4,​4'-​bipyridinium chloride iodide* was prepared by reacting 1.05 g of *1-(2,4-dinitrophenyl)-4-(pyridin-4-yl)pyridinium chloride*^[2]^ (2.9 mmol) and 1 mL of iodomethane (16.1 mmol) in 25 mL of acetonitrile at room temperature for 60 h. Then the red solid was filtered, washed with acetonitrile and dried under reduced pressure to yield pure 1-​(2,​4-​dinitrophenyl)​-​1'-​methyl-4,​4'-​bipyridinium chloride iodide as a red powder. Mass: 1.28 g, yield: 88%.

^1^H NMR (400 MHz, DMSO-D6) δ 9.72 (d, *J* = 6.9 Hz, 2H), 9.37 (d, *J* = 6.7 Hz, 2H), 9.18 (d, *J* = 2.4 Hz, 1H), 9.10 (d, *J* = 6.9 Hz, 2H), 9.04 (dd, *J* = 8.7, 2.5 Hz, 1H), 8.90 (d, *J* = 6.7 Hz, 2H), 8.47 (d, *J* = 8.7 Hz, 1H), 4.48 (s, 3H).

*Synthesis of 2-​[2-​(2-​butoxyethoxy)​ethoxy]​-​ethanol-1-​(4-​methylbenzenesulfona​te)*

Compound *2-​[2-​(2-​butoxyethoxy)​ethoxy]​-​ethanol-1-​(4-​methylbenzenesulfona​te)* was prepared by mixing 11.7 g of tri(ethylene glycol) monobutyl ether (56.8 mmol) and 10.8 g of 4-toluenesulfonyl chloride (56.7 mmol) in 300 mL of CH_2_Cl_2_ and 15 mL of NEt_3_ (112 mmol) at 0°C. Then the reaction was conducted at room temperature overnight. The mixture was concentrated to 100 mL under reduced pressure and filtered. The product was purified by column chromatography (EtOAc/pentane 1/1) on silica gel to yield a colorless oil. Mass: 13.5 g, yield: 68%.

^1^H NMR (300 MHz, CDCl_3_) δ 7.82 (d, *J* = 8.3 Hz, 2H), 7.36 (d, *J* = 8.1 Hz, 2H), 4.18 (t, *J* = 4.8 Hz, 2H), 3.70 (t, *J* = 5.1 Hz, 2H), 3.65 – 3.55 (br m, 8H), 3.46 (t, *J* = 6.7 Hz, 2H), 2.46 (s, 3H), 1.58 (tt, *J* = 14.9, 6.9 Hz, 2H), 1.37 (tq, *J* = 7.1, 4.5 Hz, 2H), 0.92 (t, *J* = 7.3 Hz, 3H).

***9/ Synthesis and characterizations of VP-A*.**

*Synthesis of 1-iodo,2-(2-​[2-​(2-​butoxyethoxy)​ethoxy]​-​ethoxy)benzene*

Compound *1-iodo,2-(2-​[2-​(2-​butoxyethoxy)​ethoxy]​-​ethoxy)benzene* was prepared by mixing 1.86 g of *2-​[2-​(2-​butoxyethoxy)​ethoxy]​-​ethanol-1-​(4-​methylbenzenesulfona​te)* (5.15 mmol), 1.05 g of 2-Iodophenol (4.8 mmol) and 2 g of K_2_CO_3_ (14.5 mmol) in 40 mL of CH_3_CN before refluxing for 14 h under argon. The mixture was filtered, and all volatiles were removed under reduced pressure. The product was purified by column chromatography (pentane/EtOAc 3/1) on silica gel, yielding a pale-yellow oil. Mass: 1.49 g, yield: 79%.

^1^H NMR (300 MHz, CDCl_3_) δ 7.76 (dd, *J* = 7.8, 1.6 Hz, 1H), 7.28 (td, *J* = 8,2, 1.6 Hz, 1H), 6.83 (dd, *J* = 8.2, 1.2 Hz, 1H), 6.70 (td, *J* = 7.6, 1.3 Hz, 1H), 4.16 (t, *J* = 5.4 Hz, 2H), 3.92 (t, *J* = 5.4 Hz, 2H), 3.80 (dd, *J* = 5.8, 3.5 Hz, 2H), 3.72 – 3.62 (m, 4H), 3.58 (tt, *J* = 4.7, 2.4 Hz, 2H), 3.45 (t, *J* = 6.7 Hz, 2H), 1.61 – 1.50 (m, 2H), 1.42 – 1.28 (m, 2H), 0.92 (t, *J* = 7.5 Hz, 3H).

^13^C NMR (75 MHz, CDCl_3_) δ 157.6 (s, *C*-I), 139.5 (s, *C*H-Ar), 129.4 (s, *C*H-Ar), 122.5 (d, *C*H-Ar), 112.6 (s, *C*H-Ar), 86.7 (s, *C*-O), 71.2 (s, -*C*H_2_-), 71.2 (s, -*C*H_2_-), 70.7 (s, -*C*H_2_-), 70.7 (s, -*C*H_2_-), 70.1 (s, -*C*H_2_-), 69.5 (s, -*C*H_2_-), 69.2 (s, -*C*H_2_-), 31.7 (s, -CH_2_-*C*H_2_-CH_2_-), 19.3 (s, -CH_2_-*C*H_2_-CH_3_), 13.9 (s, -CH_3_).

*Synthesis of* *1-(p-ethynyl-aniline)-2-(2-​[2-​(2-​butoxyethoxy)​ethoxy]​-​ethoxy)benzene*

Compound *1-(p-ethynyl-aniline)-2-(2-[2-(2-butoxyethoxy)ethoxy]-ethoxy)benzene* was prepared by mixing 1.40 g of *1-iodo,2-(2-​[2-​(2-​butoxyethoxy)​ethoxy]​-​ethoxy)benzene* (3.6 mmol), 420 mg of p-ethynyl-aniline (3.6 mmol) and 5 mL of NEt_3_ in 30 mL of THF under argon. To this solution was added 40 mg of Pd(PPh_3_)_4_ (0.04 mmol) and 100 mg of CuI (0.5 mmol) under argon and the reaction was conducted at 60 °C for 14 h. After reaction, the mixture was filtered and the product was purified by column chromatography on silica gel using pentane/EtOAc (1/4 and 1% of NEt_3_), yielding a pale yellow oil. Mass: 315 mg, yield: 23%.

^1^H NMR (300 MHz, DMSO-D6) δ 7.39 (dd, *J* = 7.6, 1.7 Hz, 1H), 7.28 (ddd, *J* = 9.1, 7.6, 1.7 Hz, 1H), 7.17 (d, *J* = 9.0 Hz, 2H), 7.03 (d, *J* = 8.0 Hz, 1H), 6.94 (td, *J* = 7.5, 0.8 Hz, 1H), 6.58 (d, *J* = 9.0 Hz, 2H), 5.48 (s, 2H), 4.16 (t, *J* = 4.2 Hz, 2H), 3.82 (t, *J* = 4.2 Hz, 2H), 3.69 (t, *J* = 4.5 Hz, 2H), 3.56 – 3.31 (m, 6H, overlapped with water traces), 2.51 (dt, *J* = 3.5, 1.8 Hz, 2H), 1.51 – 1.38 (m, 2H), 1.36 – 1.21 (m, 2H), 0.85 (t, *J* = 7.3 Hz, 3H).

^13^C NMR (75 MHz, DMSO-D6) δ 159.1 (s, *C*_IV_), 149.7 (s, *C*_IV_), 132.8 (*C*H-Ar, overlap of 2 signals), 129.5 (s, *C*H-Ar), 121.1 (s, *C*H-Ar), 114.1 (s, *C*H-Ar), 113.5 (s, *C*_IV_), 112.9 (s, *C*H-Ar), 109.4 (s, C_IV_), 95.4 (s, *C*_IV_), 83.6 (s, C_IV_), 70.8 (s, -*C*H_2_-), 70.5 (s, -*C*H_2_-), 70.4 (s, -*C*H_2_-), 70.3 (s, -*C*H_2_-), 69.9 (s, -*C*H_2_-), 69.5 (s, -*C*H_2_-), 68.8 (s, -*C*H_2_-), 31.8 (s, -*C*H_2_-), 19.3 (s, -*C*H_2_-), 14.2 (s, -*C*H_3_).

*Synthesis of 1-methyl-4,4'-bipyridinium-1’-(p-ethynyl-benzyl)-2-(2-[2-(2-butoxyethoxy)ethoxy]-ethoxy)benzene* (**VP-A**)

A solution containing *1-(p-ethynyl-aniline)-2-(2-[2-(2-butoxyethoxy)ethoxy]-ethoxy)benzene* (160 mg, 0.4 mmol) and *1-​(2,​4-​dinitrophenyl)​-​1'-​methyl-4,​4'-​bipyridinium chloride iodide* (196 mg, 0.4 mmol) in MeOH (15 mL) and DMSO (3 mL) was stirred at 70°C for 14 h under argon. Then all volatiles were removed and the residue was precipitated with 50 mL of EtOAc. The pink solid was filtered and washed with EtOAc to remove 2,​4-​dinitro-benzenamine. The solid was dissolved in 5 mL of demineralized water before addition of 5 mL of a KPF_6_ (300 mg) aqueous solution to precipitate a red salt. After filtration, the red solid was washed with demineralized water and the full anion exchange was performed via a suspension of the red PF_6_^-^ salt in demineralized water in the presence of Amberlite® IRA900 Cl for 14 h under slow magnetic stirring (< 100 rpm) at room temperature. The red solution was filtered and after elimination of water under reduced pressure, a red solid was obtained. Mass: 140 mg, yield: 58%.

HRMS (ESI) calcd for [C_35_H_40_N_2_O_4_]^2+^: *m/z* = 276.1489; found 276.1483.

^1^H NMR (400 MHz, D_2_O) δ 9.40 (d, *J* = 6.6 Hz, *H5*, 2H), 9.10 (d, *J* = 6.5 Hz, *H2*, 2H), 8.61 (d, *J* = 6.6 Hz, *H4*, 2H), 8.52 (d, *J* = 6.5 Hz, *H3*, 2H), 7.90 (d, *J* = 8.5 Hz, *H6*, 2H), 7.73 (d, *J* = 8.5 Hz, *H7*, 2H), 7.43 (dd, *J* = 6.8, 1.2 Hz, *H11*, 1H), 7.22 (t, *J* = 7.8 Hz, *H9*, 1H), 7.01 – 6.92 (m, overlapped signals of *H8* and *H10*, 2H), 4.55 (s, *H1*, 3H), 4.31 (s, -OC*H*_2_-, 2H), 4.07 (s, -OC*H*_2_-, 2H), 3.96 – 3.89 (m, -OC*H*_2_-, 2H), 3.75 (d, *J* = 4.2 Hz, -OC*H*_2_-, 2H), 3.64 – 3.58 (m, -OC*H*_2_-, 2H), 3.53 – 3.45 (m, -OC*H*_2_-, 2H), 3.37 (t, *J* = 6.8 Hz, *H18*, 2H), 1.44 – 1.34 (m, *H19*, 2H), 1.19 (dq, *J* = 14.6, 7.3 Hz, *H20*, 2H), 0.78 (t, *J* = 7.4 Hz, *H21*, 3H).

^13^C NMR (101 MHz, D_2_O) δ 158.6 (*C*_IV_), 150.0 (*C*_IV_), 148.5 (*C*_IV_), 146.4 (*C*H*2*), 144.7 (*C*H*5*), 141.2 (*C*_IV_), 133.7 (*C*H*11*), 133.4 (*C*H*7*), 131.5 (*C*H*9*), 126.6 (*C*H*4*), 126.5 (*C*H*3*), 126.3 (*C*_IV_), 124.2 (*C*H*6*), 121.6 (*C*H*10*), 112.7 (*C*H*8*), 110.6 (*C*_IV_), 92.1 (*C*_IV_), 89.2 (*C*_IV_), 70.82 (*C*H*_18_*), 70.3 (O*C*H_2_), 69.8 (O*C*H_2_), 69.6 (O*C*H_2_), 69.3 (O*C*H_2_), 69.1 (O*C*H_2_), 68.4 (O*C*H_2_), 48.5 (*C*H*1*), 30.7 (*C*H*19*), 18.5 (*C*H*20*), 13.1 (*C*H*21*).

***Figure S3***. ^1^H NMR spectrum of **VP-A** in D_2_O (400 MHz, 298 K).

***Figure S4***. ^13^C -APT NMR spectrum of **VP-A** in D_2_O (101 MHz, 298 K).

***Figure S5***. ^1^H-^13^C HMQC NMR spectrum of **VP-A** in D_2_O (400 MHz, 298 K).

***10/ Preparation and NMR spectra of the VP-A-PV•CB[8]_4_ complex.***

A solution of **VP-A-PV**•CB[8]_4_ was prepared from a mixture of 0.80 mg of solid CB[8], 31 µL of a 5 mM stock solution of **VP-A-PV**•Cl_4_ and D_2_O for a final concentration of 1 mM of CB[8] (corresponding to 0.25 mM of **VP-A-PV**•CB[8]_4_). Acetone was used as internal reference (2.22 ppm). A ratio of 4.0 CB[8] for 1 **VP-A-PV** was calculated based on integrals of protons e at 9.22 ppm (4H) and integrals of protons of CB[8] at 5.78 ppm (64H). A long relaxation delay (“d1”) of 60 s was applied to confirm that the disappearance of “h” signal is not due to a long relaxation time and get reliable integral values.

^1^H NMR (400 MHz, D_2_O) δ 9.22 (s, *He*, 4H), 8.86 (s, *Hb*, 4H), 8.17 (d, *J* = 7.5 Hz, *Hf* or *Hg*, 4H), 8.04 (s, *Hf* or *Hg*, 4H), 7.74 (s, *Hd*, 4H), 7.58 (s, *Hc*, 4H), 5.78 (d, *J* = 14.3 Hz, *HCB[8]*, 64H), 5.53 (s, *HCB[8]*, 64H), 4.42 (s, *Ha*), 4.22 (d, *J* = 14.8 Hz, *HCB[8]*, 64H), 3.98-3.10 (m, -OC*H_2_*-), 2.22 (ref acetone), 0.88 (s, *Hp-q*, 8H), 0.36 (s, *Hr*, 6H).

***Figure S6***. ^1^H NMR spectrum of **VP-A-PV**•CB[8]_4_ at 300 K (500 MHz, D_2_O, complex conc. 0.25 mM).

***Figure S7***. ^1^H-^1^H COSY NMR spectrum (400 MHz) of **VP-A-PV•**CB[8]_4_ (D_2_O, 298 K, conc. 0.25 mM).

***11/ Preparation and NMR spectra of the* VP-A•CB[8]_2_ complex.**

A 0.5 mM solution of **VP-A**•CB[8]_2_ was prepared by addition of 59 µL of a **VP-A** stock solution at 5 mM in D_2_O and 536 µL of D_2_O onto 0.79 mg of solid CB[8] (final concentration of CB[8]: 1 mM, corresponding to a complex concentration of 0.5 mM).

^1^H NMR (300 MHz, D_2_O) δ 9.24 (d, *J* = 6.5 Hz, *He*, 2H), 8.84 (d, *J* = 5.7 Hz, *Hb*, 2H), 8.17 (d, *J* = 8.4 Hz, *Hf*, 2H), 8.04 (d, *J* = 8.6 Hz, *Hg*, 2H), 7.69 (d, *J* = 6.0 Hz, *Hd*, 2H), 7.48 (d, *J* = 6.1 Hz, *Hc*, 2H), 7.39 – 7.30 (br s, 1H), 7.11 (br s, 1H), 6.97 (br s, 1H), 5.78 (d, *J* = 15.3 Hz, CB[8], 28H), 5.52 (s, CB[8], 28H), 4.57 (s, *Ha*), 4.39 (s, -OC*H_2_*-, 2H), 4.22 (d, *J* = 15.3 Hz, CB[8], 28H), 4.06 (d, *J* = 17.2 Hz, -OC*H_2_*-, 2H), 3.83 (br s, -OC*H_2_*, 2H), 3.57 (br s, -OC*H_2_*-, 2H), 3.32 (br m, -OC*H_2_*-, 4H), 3.04 (t, *J* = 6.8 Hz, *Hr*, 2H), 2.22 (acetone, ref), 0.81 (br m, overlapped signals of *Hs* and *Ht*, 4H), 0.37 (t, *J* = 7.0 Hz, *Hu*, 3H).


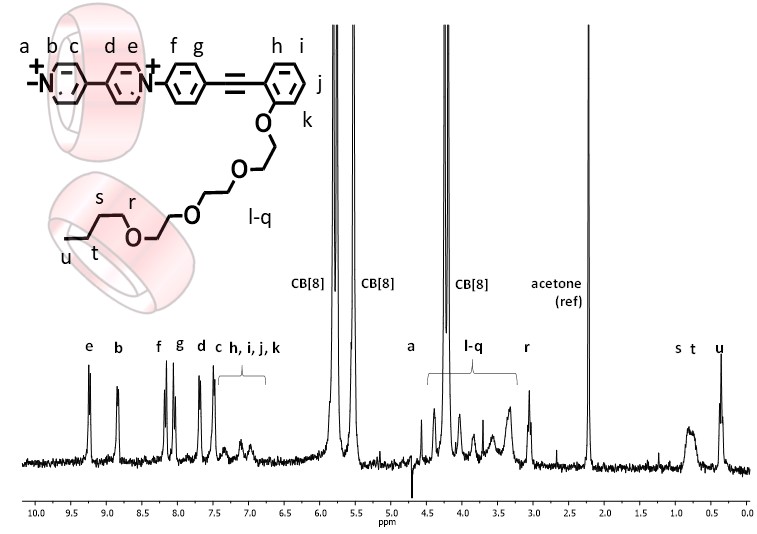


***Figure S8***. ^1^H NMR spectrum of **VP-A•**CB[8]_2_ in D_2_O (300 MHz, 298 K, complex conc 0.5 mM).


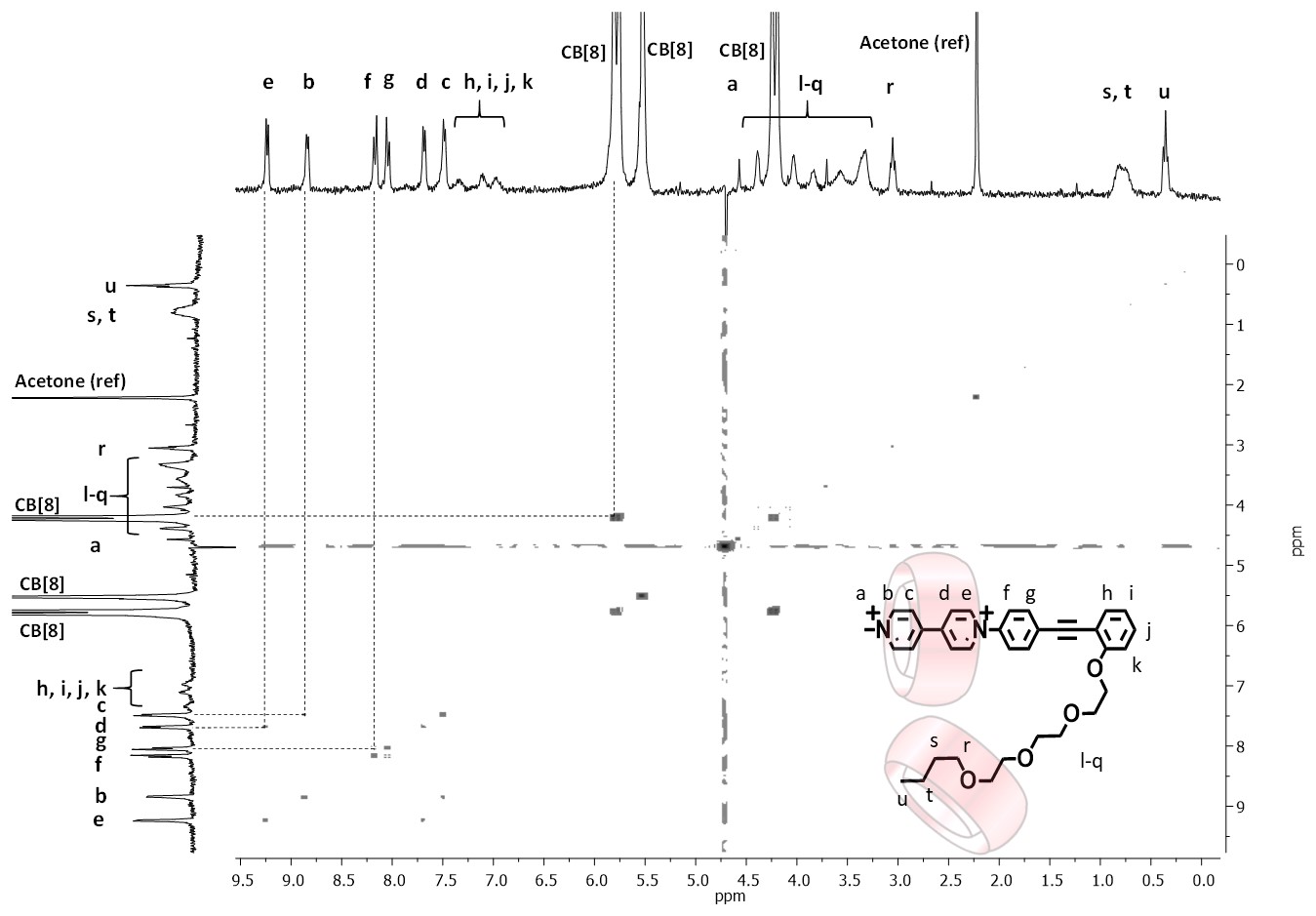


***Figure S9***. ^1^H-^1^H COSY NMR spectrum of **VP-A•**CB[8]_2_ in D_2_O at 298 K (complex conc. 0.5 mM).

***Figure S10***. ^1^H NMR spectrum of **VP-A**•CB[8]_2_ at 300 K (500 MHz, D_2_O, complex conc. 0.5 mM). Ratio of integrals are in line with 1 **VP-A** for 1.75 CB[8].

***12/ Variable temperature NMR spectra of VP-A-PV*.**


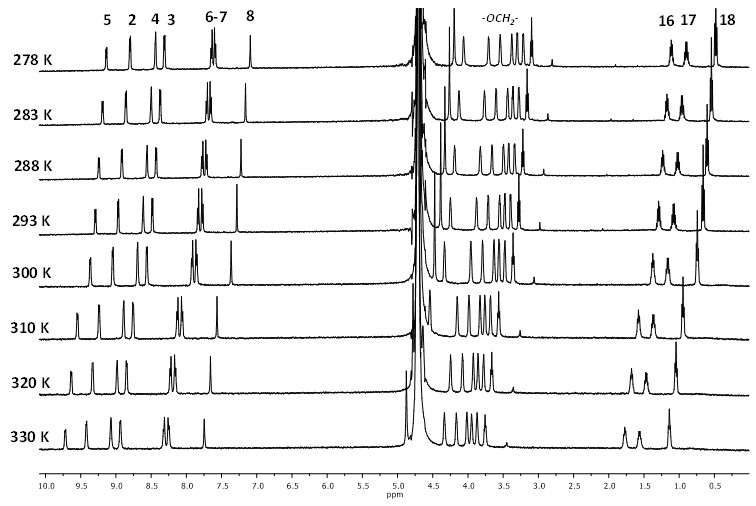


***Figure S11***. Variable temperature ^1^H NMR spectra of **VP-A-PV** (D_2_O, 500 MHz).

***13/ 500 MH zand 800 MHz variable temperature NMR spectra of the VP-A-PV●CB[8]_4_ complex*.**


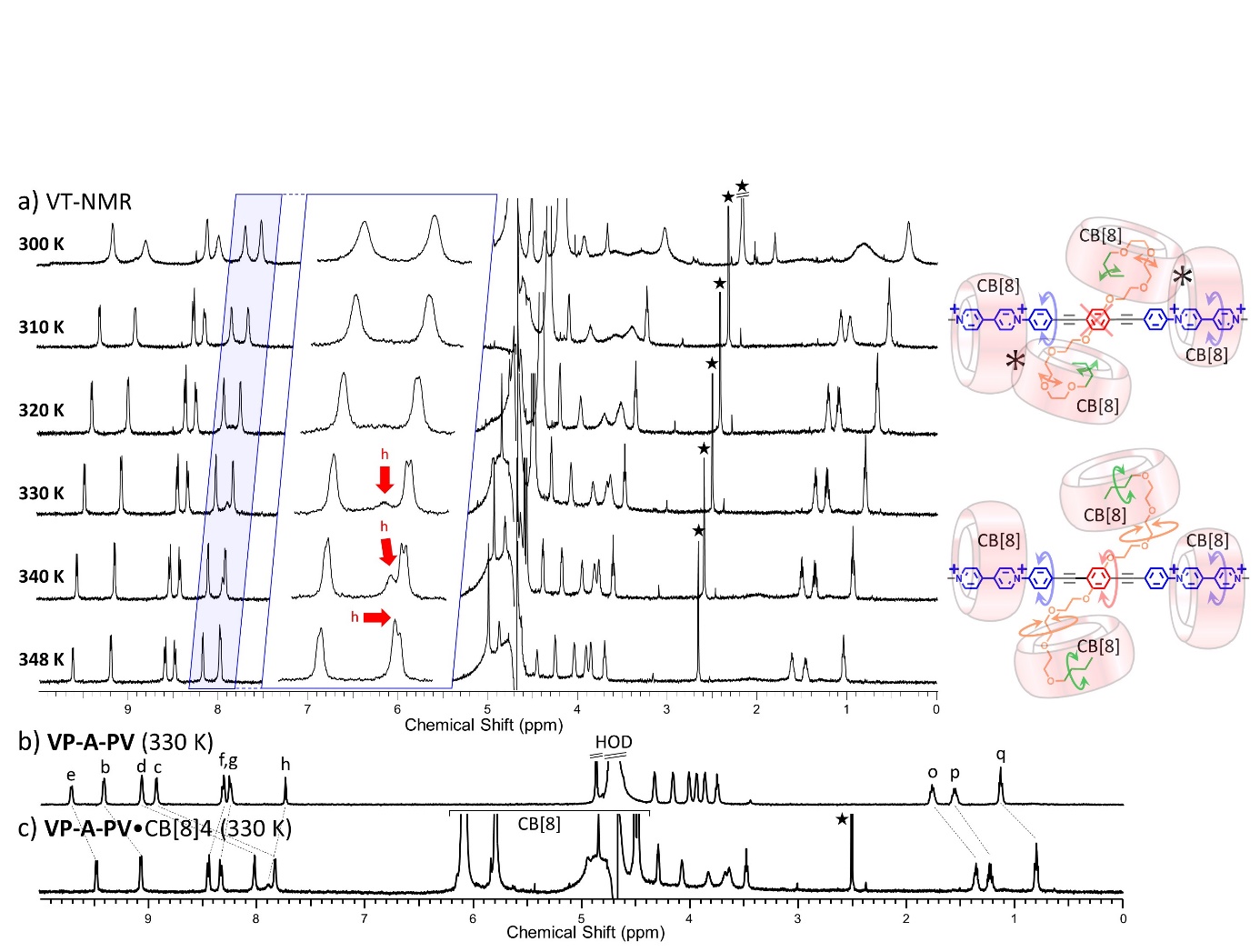


***Figure S12***. Variable temperature ^1^H NMR spectra of **VP-A-PV**•CB[8]_4_ (500 MHz, D_2_O, 0.25 mM).

***Figure S13***. Variable temperature ^1^H NMR spectra of **VP-A-PV**•CB[8]_4_ (800 MHz, D_2_O, 0.25 mM). Small additional peaks in the aromatic region at low temperatures are assigned to signals belonging to a new complex presumably due to possible CB[8] precipitation below 10°C.

***14/ NMR tests of HO-(EG)_3_-Bu binding by CB[7] and CB[8]*.**

***Figure S14***. ^1^H NMR spectra (300 MHz, 298 K, D_2_O) of (a) tri(ethylene glycol) monobutyl ether: HO-(EG)_3_-Bu, (b) HO-(EG)_3_-Bu with 0.5 equiv. of CB[7] and (c) HO-(EG)_3_-Bu with 0.5 equiv. of CB[8].

***15/ Superimposed structures from the MD trajectory of the complex VP-A-PV•CB[8]_4_.***


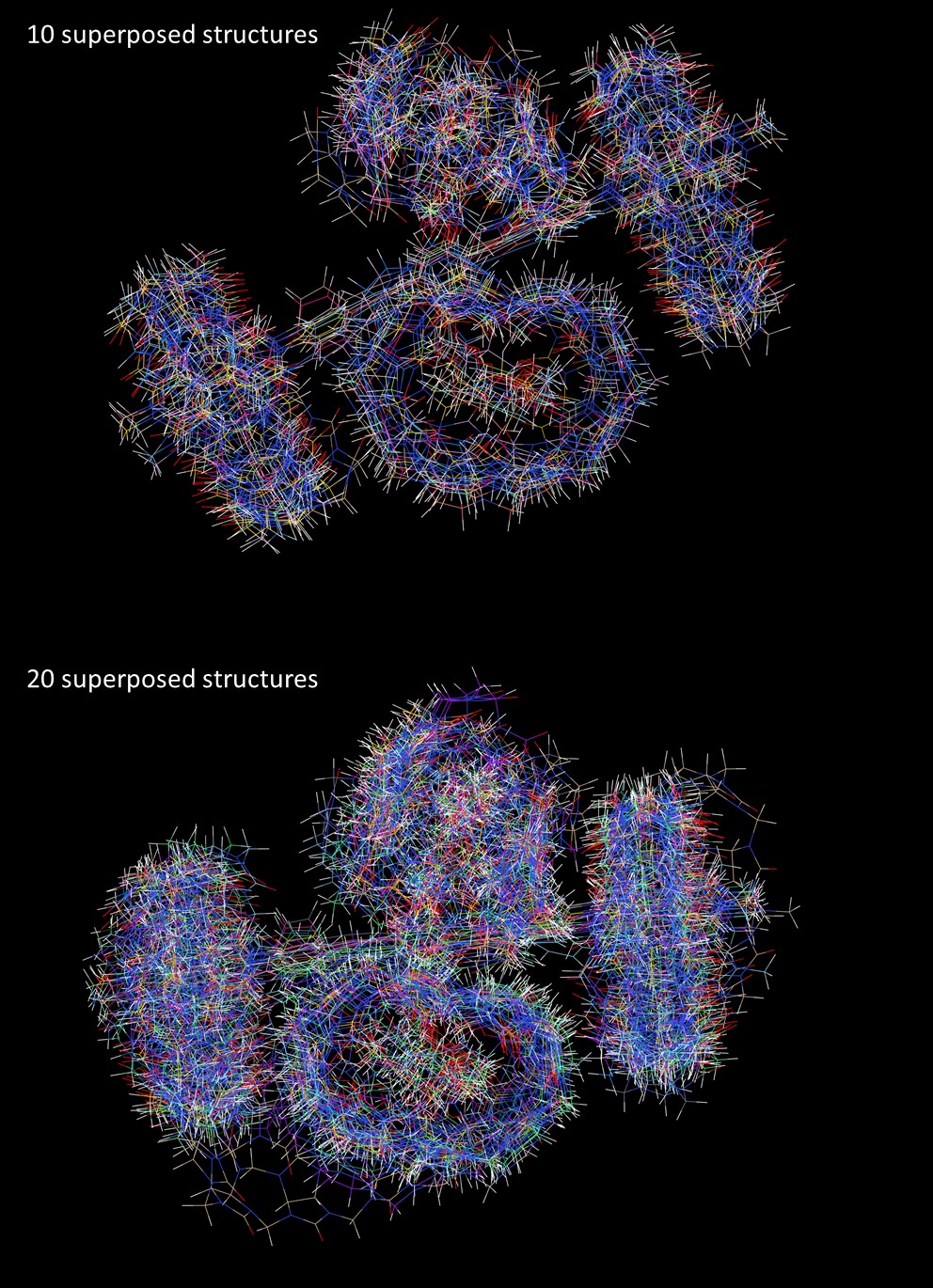


***Figure S15***. Superimposed structures of sets of frames from the molecular dynamic simulation corresponding to **VP-A-PV*•***CB[8]_4_ in water.

***16/ Variable temperature NMR spectra of VP-A•CB[8]_2_.***

***
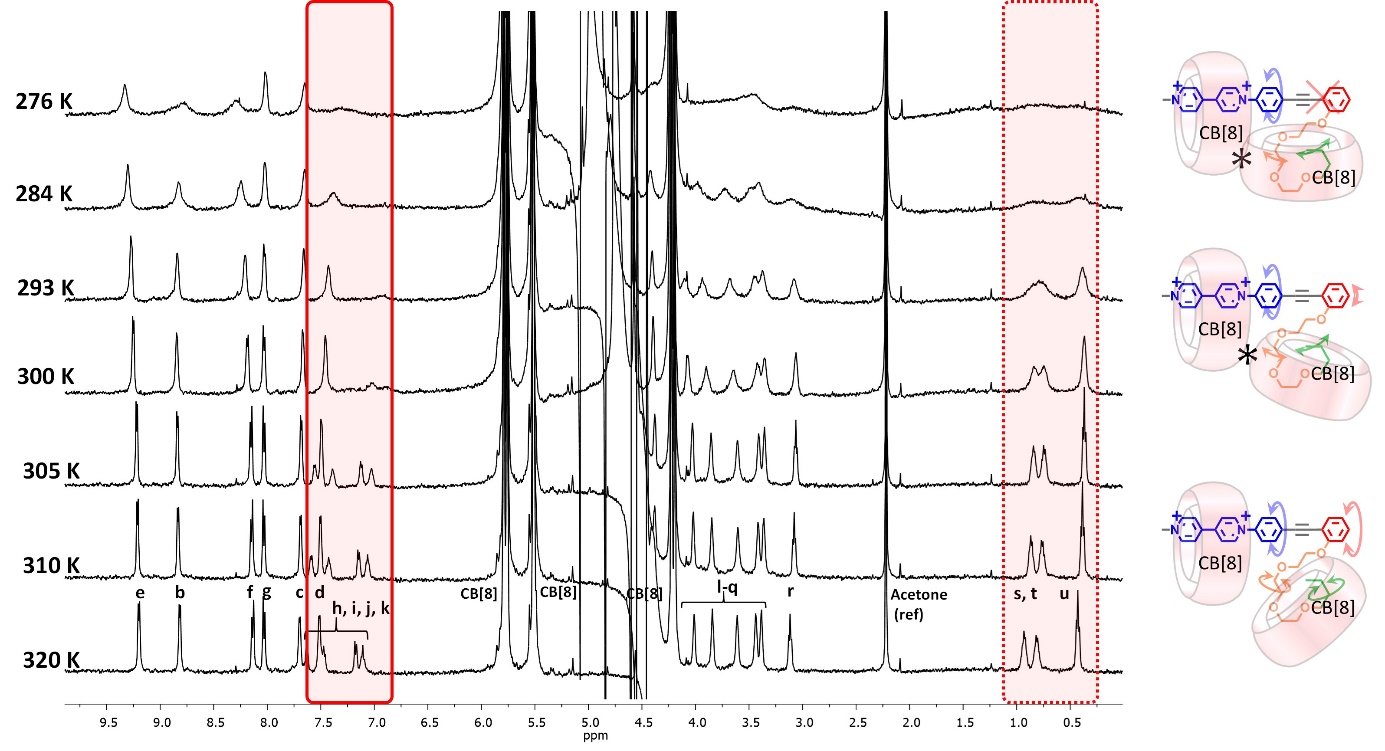
***

***Figure S16***. Variable temperature ^1^H NMR spectra (500 MHz, D_2_O) of the **VP-A**•CB[8]_2_ complex showing the gradual disappearance of signals corresponding to phenyl protons “h, i, j, k” upon cooling near 300 K (red rectangle, left) followed by the very large broadening of signals assigned to the butyl in CB[8] near 284 K (red rectangle, right). At high temperatures, all signals are recovered.

***17/ Preparation, NMR spectra and ITC of the VP-A•CB[7] complex.***

A 1.0 mM solution of **VP-A**•CB[7] was prepared by addition of 100 µL of a **VP-A** stock solution at 5 mM in D_2_O, 100 µL of a CB[7] stock solution at 5 mM (prepared by dissolution of 58.15 mg in 5.0 mL of D_2_O) and 300 µL of D_2_O, leading to an orange solution.

^1^H NMR (300 MHz, D_2_O) δ 9.32 (d, *J* = 6.5 Hz, *He*, 2H), 8.98 (d, *J* = 6.2 Hz, *Hb*, 2H), 8.25 (d, *J* = 8.4 Hz, *Hf*, 2H), 8.03 (d, *J* = 8.4 Hz, *Hg*, 2H), 7.68 (dd, *J* = 7.1, 1.5 Hz, *Hk*, 1H), 7.51 (t, *J* = 7.9 Hz, *Hi*, 1H), 7.30 – 7.11 (m, overlapped signal of *Hd*, *Hh* and *Hj*, 4H), 7.02 (br s, *Hc*, 2H), 5.68 (m, CB[7], 14H), 5.50 (s, CB[7], 14H), 4.38 (br t, *J* = 4.3 Hz, -OC*H_2_*-, 2H), 4.24 (m, CB[7], 14H), 4.03 (br t, *J* = 4.3 Hz, -OC*H_2_*-, 2H), 3.93 – 3.84 (m, -OC*H_2_*-, 2H), 3.73 – 3.68 (m, -OC*H_2_*-, 2H), 3.66 – 3.60 (m, -OC*H_2_*-, 2H), 3.59 – 3.52 (m, -OC*H_2_*-, 2H), 3.45 (t, *J* = 6.7 Hz, *Hr*, 2H), 2.22 (ref acetone), 1.52 – 1.40 (m, *Hs*, 2H), 1.26 (dt, *J* = 14.7, 7.3 Hz, *Ht*, 2H), 0.83 (t, *J* = 7.3 Hz, *Hu*, 3H).

***Figure S17***. a. ^1^H NMR spectrum of **VP-A•**CB[7]**,** b. ^1^H-^1^H COSY NMR spectrum of **VP-A•**CB[7] in D_2_O (300 MHz, 298 K, 1 mM).


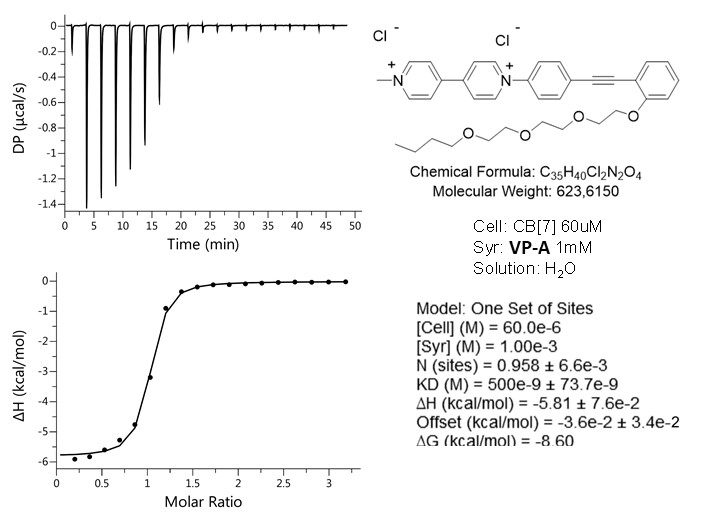


***Figure S18***. isothermal titration calorimetry (ITC) experiment with **VP-A** and CB[7].

***Figure S19***. ^1^H NMR spectrum of **VP-A•**CB[7] in presence of 2 equiv. of CB[8] in D_2_O (300 MHz, 298 K).

***18/ Preparation, NMR spectra and ITC of the VP-A-PV•CB[7]_2_ complex.***

A 0.77 mM solution of **VP-A-PV**•CB[7]_2_ was prepared by adding 400 μL of a **VP-A-PV**•Cl_4_ stock solution at 1 mM in D_2_O (prepared by dissolution of 11.70 mg in 10.0 mL of D_2_O) to 80 μL of a CB[7] stock solution at 10 mM (prepared by dissolution of 58.15 mg in 5.0 mL of D_2_O) and 40 μL of D_2_O, leading to an orange solution.

^1^H NMR (400 MHz, D_2_O) δ 9.27 (d, *J* = 5.8 Hz, *He*, 4H), 8.94 (d, *J* = 6.2 Hz, *Hb*, 4H), 8.21 (d, *J* = 7.5 Hz, *Hf* or *Hg*, 4H), 7.99 (d, *J* = 8.4 Hz, *Hf* or *Hg*, 4H), 7.40 (s, *Hh*, 2H), 7.08 (d, *J* = 6.5 Hz, *Hd*, 4H), 6.96 (d, *J* = 6.2 Hz, *Hc*, 4H), 5.63 (overlapped doublets, *J* = 14.5, 15.1 Hz, *HCB[7]*, 28H), 5.46 (s, *HCB[7]*, 28H), 4.59 (s, *Ha*), 4.34 (br m, -OC*H_2_*-, 4H), 4.18 (overlapped doublets, *J* = 15.1, 15.6 Hz, *HCB[7]*, 28H), 3.97 (br m, -OC*H_2_*-, 4H), 3.82 (br m, -OC*H_2_*-, 4H), 3.64 (br m, -OC*H_2_*-, 4H), 3.56 (br m, -OC*H_2_*-, 4H), 3.48 (br m, -OC*H_2_*-, 4H), 3.36 (br m, -OC*H_2_*-, 4H), 3.27 (br m, -OC*H_2_*-, 4H), 2.22 (ref acetone), 1.38 (br m, *Hp*, 4H), 1.16 ( br m, *Hq*, 4H), 0.75 (br m, *Hr*, 6H).


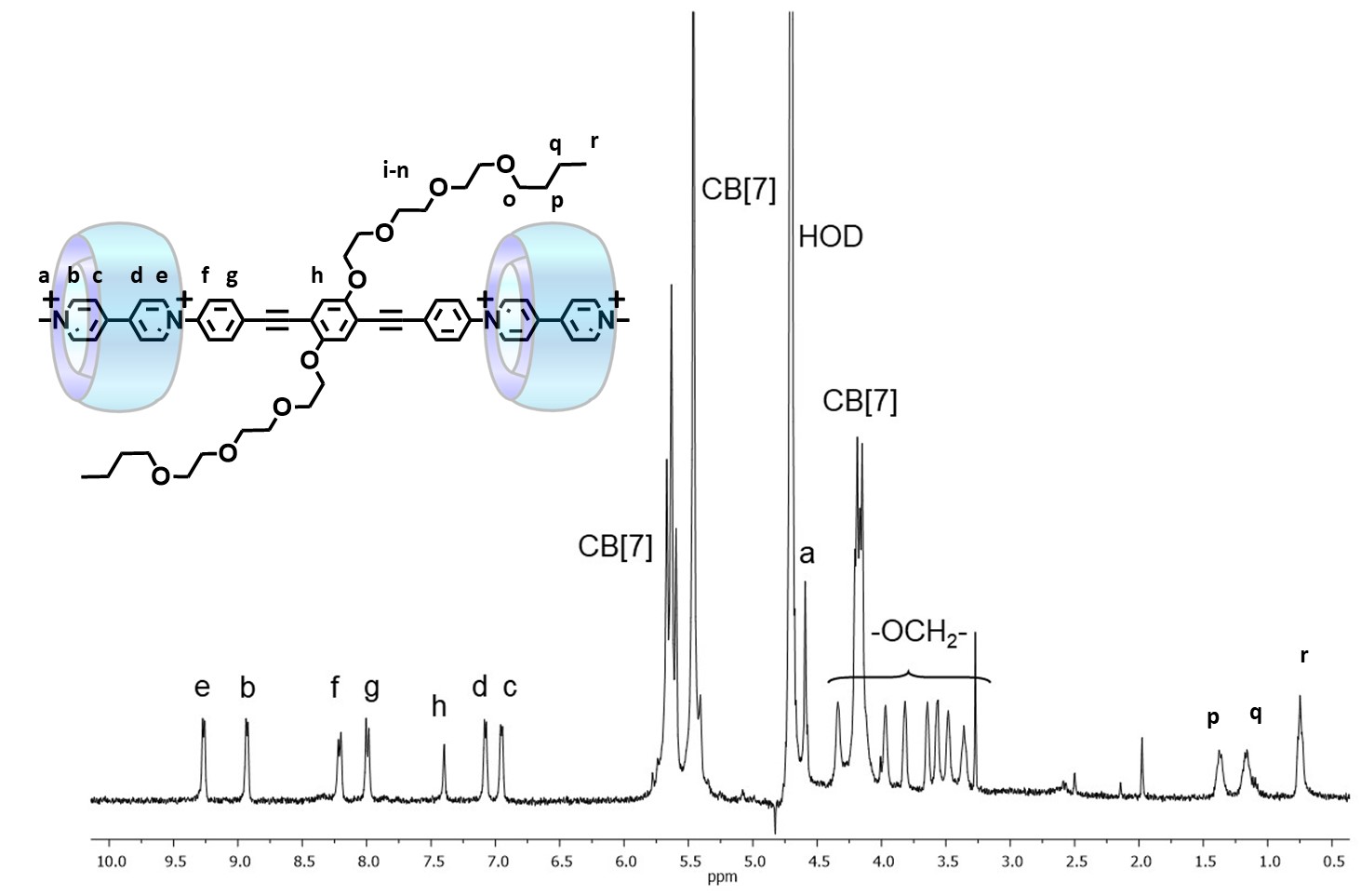


***Figure S20*** ^1^H NMR spectrum of **VP-A-PV**•CB[7]_2_ in D_2_O (complex conc 0.77 mM).


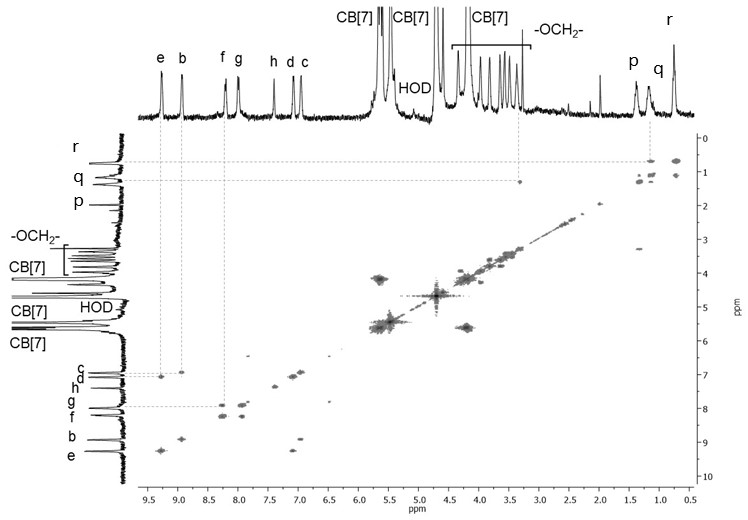


***Figure S21.*** ^1^H-^1^H COSY NMR spectrum of **VP-A-PV**•CB[7]_2_ in D_2_O (complex conc 0.77 mM).


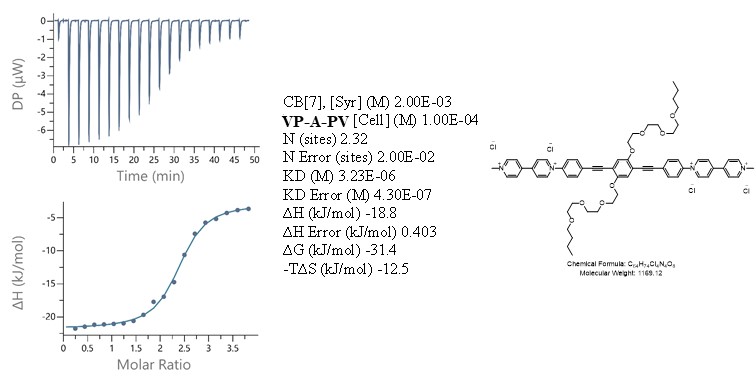


***Figure S22***. Isothermal titration calorimetry (ITC) experiment with **VP-A-PV** and CB[7].

***19/ Preparation and NMR spectra of the “hybrid” VP-A-PV•CB[7]_2_•CB[8]_2_ complex.***

Titration of **VP-A-PV*•***CB[7]_2_ with CB[8]: each tube was prepared independently via addition of 124-37 µL of a 4 mM stock solution of **VP-A-PV** (5.0 10^-7^- 1.5 10^-7^ mol), 155-37 µL of a 12 mM stock solution of CB[7] (1.5 10^-6^ – 4.4 10^-7^ mol) and 215-505 µL of D_2_O added onto 0.66-0.78 mg of solid CB[8] (5.0 10^-7^-5.9 10^-7^ mol). **VP-A-PV**/CB[7]/CB[8] ratios of 1/3/1, 1/3/2, 1/3/3 and 1/3/4 were obtained, respectively. The CB[8] concentration was 1 mM in each solution. A 1/3 ratio for **VP-A-PV**/CB[7] was selected since **VP-A-PV**/CB[7] 1/2 ratios led to mixtures of **VP-A-PV*•***CB[8]_4_ and **VP-A-PV*•***CB[7]_2_•CB[8]_2_. However, a low concentration of the **VP-A-PV*•***CB[8]_4_ complex could still be observed in 1/3/3 and 1/3/4 **VP-A-PV**/CB[7]/CB[8] solutions (Figure 6 of the paper).

*1/3/3* ***VP-A-PV****/CB[7]/CB[8] solution at 298 K (Figure S23) :* ^1^H NMR (800 MHz, D_2_O, **298 K**) δ 9.41 (s, *J* = 5.1 Hz, *He*, 4H), 9.25 (s, *He_CB[8]_*, 0.4H), 9.06 (s, *Hb*, 4H), 8.89 (s, *Hb_CB[8]_*, 0.4H), 8.38 (s, *Hf-g*, 4H), 8.24 (s, *Hf-g_CB[8]_*, 0.4H), 8.07 (s, *Hf-g*, 4H), 7.78 (s, *Hd_CB[8]_*, 0.4H), 7.62 (s, *Hc_CB[8]_*, 0.4H), 7.20 (d, *J* = 6.1 Hz, *Hd*, 4H), 7.07 (d, *J* = 5.8 Hz, *Hc*, 4H), 6.00 – 5.62 (m, *HCB[7]+HCB[8]*, 102H), 5.62 – 5.35 (m, *HCB[7]+HCB[8]*, 99H), 4.72 (s, *Ha*), 4.50 (s, -OC*H_2_*-, 4H), 4.37 – 4.15 (m, *HCB[7]+HCB[8]*, 100H), 4.05 (s, -OC*H_2_*-, 4H), 3.66 (s, -OC*H_2_*-, 4H), 3.14 (s, -OC*H_2_*-, 16H), 2.22 (acetone, ref), 0.99 (s, *Hpq*, 8H), 0.42 (s, *Hr*, 6H).

*1/3/3* ***VP-A-PV****/CB[7]/CB[8] solution at 350 K (Figure S24) :* ^1^H NMR (800 MHz, D_2_O, **350 K**) δ 9.34 (s, 4H), 9.02 (s, 4H), 8.31 (s, 4H), 8.10 (s, 4H), 7.70 (s, 2H), 7.21 (s, 4H), 7.07 (s, 4H), 5.94-5.79 (m, *HCB[7]+HCB[8]*, 97H), 5.70 – 5.47 (m, *HCB[7]+HCB[8]*, 94H), 4.71 (s, 6H), 4.49 (t, *J* = 4 Hz, 4H), 4.40 – 4.15 (m, *HCB[7]+HCB[8], signals affected by water suppression*), 4.05 (t, *J* = 4.8 Hz, 4H), 3.83 (s, 4H), 3.61 (s, 4H), 3.42 (m, 12H), 2.23 (acetone, ref), 1.36 (s, 4H), 1.19 (s, 4H), 0.75 (s, 6H).


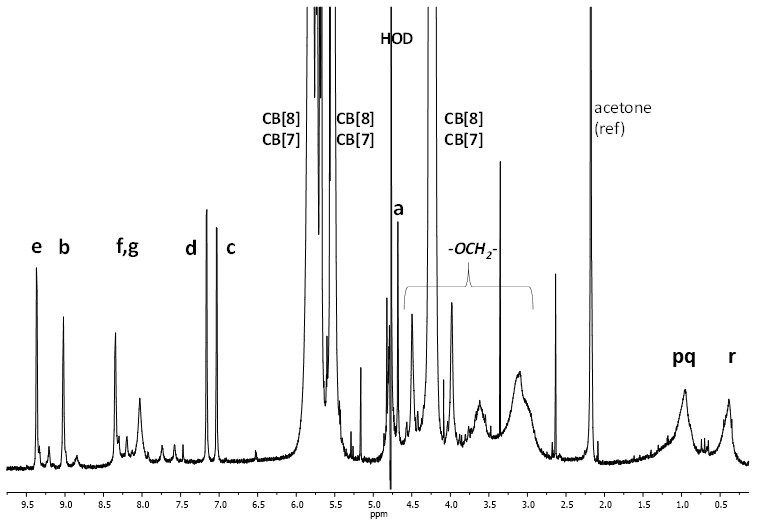


***Figure S23***. ^1^H NMR spectrum (800 MHz) of **VP-A-PV•**CB[7]_2_•CB[8]_2_ at 298 K in D_2_O (complex conc. 0.33 mM, minor signals of **VP-A-PV•**CB[8]_4_ are observed).


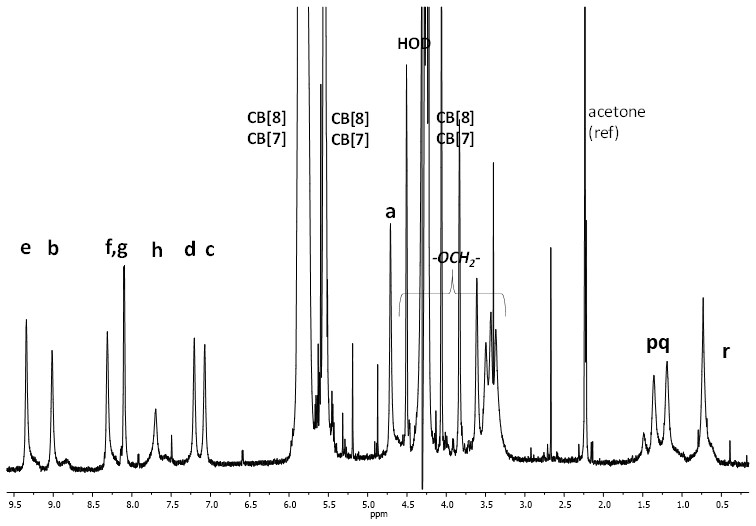


***Figure S24***. ^1^H NMR spectrum (800 MHz) of **VP-A-PV**•CB[7]_2_•CB[8]_2_ at 350 K in D_2_O (complex conc. 0.33 mM).

***Figure S25***. ^1^H-^1^H COSY NMR spectrum (800 MHz) of **VP-A-PV**•CB[7]_2_•CB[8]_2_ at 350 K in D_2_O (complex conc. 0.33 mM).

***20/ ^1^H MAS NMR spectrum of VP-A-PV•CB[7]_2_•CB[8]_2_.***

***Figure S26***. ^1^H NMR spectra of **VP-A-PV*•***CB[7]_2_•CB[8]_2_. (a) ^1^H MAS NMR spectrum of a **VP-A-PV*•***CB[7]_2_•CB[8]_2_ solution (conditions: 0.33 mM, D_2_O, 400 MHz, spinning rate 4 KHz); (b) 300 MHz ^1^H NMR spectrum of **VP-A-PV*•***CB[7]_2_•CB[8]_2_ solution (^1^H NMR analysis of the solution before ^1^H MAS NMR analysis, solution prepared from a 1/3/3 **VP-A-PV**/CB[7]/CB[8] mixture, final concentration of the complex : 0.33 mM in D_2_O).

**21/ Determination of binding constants by competition NMR.**

***Figure S27***. Competition of **VP-A-PV** and DiQuat for CB[8] inclusion; a. ^1^H NMR spectrum of **VP-A-PV**•CB[8]_4_; b. ^1^H NMR spectrum resulting for the addition 1 equiv of DiQuat into a solution of **VP-A-PV**•CB[8]_4_, leading to a mixture of **VP-A-PV**•CB[8]_4_ (almost 50%mol of total **VP-A-PV**) and **VP-A-PV**_2_•CB[8]_3_ (almost 50%mol of total **VP-A-PV**); c. ^1^H NMR spectrum of **VP-A-PV**_2_•CB[8]_3_; d. COSY NMR spectrum of **VP-A-PV**•CB[8]_4_ + 1 equiv of DQ (DiQuat).

***Figure S28***. Competition of **VP-A-PV** and DiQuat for CB[8] inclusion; a. ^1^H NMR spectrum of **VP-A-PV**•CB[7]_2_•CB[8]_2_ and 1 equiv. of DiQuat; b. Zoom of the 9.7-7.0 ppm region of the ^1^H NMR spectra of mixture of **VP-A-PV**•CB[7]_2_•CB[8]_2_ (0.5 mM) and 1 equiv. of DiQuat, of mixture of DiQuat (0.5 mM) and 2 equiv. of CB[8] and free DiQuat; 600 MHz, 300 K.

Following a previously established method,^[5]^ we analyzed by ^1^H NMR the competition of CB[8] complexation to determine the binding constant *K*_a_ of butyl groups for CB[8] in the **VP-A-PV**•CB[7]_2_•CB[8]_2_ complex using DiQuat (DQ) as competitor. DQ is featured by a binding constant *K*_a DQ⦁CB[8]_ = 4.8×10^4^ M^-1^ toward CB[8],^[6]^ and a negligible binding constant *K*_a DQ⦁CB[7]_ toward CB[7], which prefer binding the viologen groups of **VP-A-PV**.

***Competition experiments:*** solutions of DiQuat (DQ) or DQ•CB[8] (**Eq 1**) were prepared (0.5 mM in D_2_O) and the NMR spectra were recorded at 300 K on a Bruker AC600 spectrometer. The ^1^H NMR spectra permitted to determine the values of the observed chemical shifts δ_obs_ of the *H*_1_-protons of free DQ (δ_obs_DQ_ = 9.362 ppm, Figure S28) and DQ•CB[8] (δ_obs_ DQ•CB[8]_ = 8.827 ppm, Figure S28). We evaluated the chemical shift difference of the *H*_1_-protons between free DQ and DQ•CB[8] : Δδ_obs_ = δ_obs_DQ_ - δ_obs_DQ•CB[8]_ = 0.535 ppm (residual HOD signal used as internal reference).

A competitive solution (**Eq 2**) was prepared by mixing **VP-A-PV** (0.5 mM), 2 equiv of CB[7], 2 equiv of CB[8] and 1 equiv of DQ in D_2_O (residual HOD signal used as internal reference) and the ^1^H NMR spectrum (Figure S28) permitted to evaluate the values of the δ_obs_ (8.914 ppm, observed chemical shifts for *H*_1_-protons of DQ in competition, Figure S28b). The values of Δδ_obs_ and δ_obs_ were used to calculate the ratio [DQ_free_]:[DQ⦁CB[8]] here found equal to 0.16:0.84 (Eq1).

The integrals corresponding to signals of free (1.08, 10%, Figure S28a) and complexed (9.87, 90%, Figure S28a) butyl groups provided an estimation of [**VP-A-PV**•CB[7]_2_] and [**VP-A-PV**•CB[7]_2_•CB[8]_2_] species (Eq2). The equilibrium constant of the competition *K_a_* is represented in **Eq 3**. The binding constant of *K*_a DQ⦁CB[8]_ of 4.8×10^4^ M^-1^ has been determined by Kaifer et al.^[6]^

**Eq 1** DQ + CB[8] ⇄ DQ⦁CB[8] K_DQ⦁CB[8]_ = [DQ⦁CB[8]] / ([DQ]×[CB[8]])

[CB[8]] = [DQ•CB[8]] / ([DQ]× K_DQ⦁CB[8]_)

**Eq 2** **VP-A-PV**•CB[7]_2_ + 2 CB[8] ⇄ **VP-A-PV**•CB[7]_2_•CB[8]_2_

K_a_ = [**VP-A-PV**•CB[7]_2_•CB[8]_2_] / ([**VP-A-PV**•CB[7]_2_]×[CB[8]]²)

[CB[8]] = √([**VP-A-PV**•CB[7]_2_•CB[8]_2_] / ([**VP-A-PV**•CB[7]_2_]× k*_a_*))

**Eq 3** √K_a_ $=\surd( \frac{[\mathbf{VP-A-PV}\bullet CB[7]2\bullet CB[8]2]}{[\mathbf{VP-A-PV}\bullet CB[7]2]})$ × $\frac{[DQ]}{[DQ\bullet CB\left[ 8 \right]]}$ × *K*_DQ⦁CB[8]_ = √($\frac{0.10}{0.9})$ × $\frac{0.16}{0.84} \times$4.8 × 10^4^ = 3047

***K*_a_ = 9 × 10^6^ M^-2^**

These calculations assume identical CB[8] binding events for the 1^st^ and 2^nd^ complexation of butyl groups of **VP-A-PV**•CB[7]_2_ and allowed to estimate, in these conditions, the following value of *K*_a_ ≈ 9 × 10^6^ M^-2^. However, two different CB[8] binding events cannot be excluded, the first preorganizing the binding of the following, but we could not investigate this scenario of CB[8] complexation of **VP-A-PV**•CB[7]_2_ because NMR did not provide clear evidence of the intermediate species (involving only one CB[8]).

***22/ Variable temperature ^1^H NMR spectra of VP-A-PV•CB[7]_2_•CB[8]_2_.***

***Figure S29***. Variable temperature ^1^H NMR spectra of **VP-A-PV*•***CB[7]_2_•CB[8]_2_ (0.33 mM, D_2_O, 800 MHz).

***23/ ROESY spectra of VP-A-PV•CB[7]_2_•CB[8]_2_.***

***Figure S30***. ROESY NMR spectrum of **VP-A-PV•**CB[7]_2_•CB[8]_2_ (500 MHz, D_2_O, mixing time 400 ms, prepared with 1 equiv. of **VP-A-PV**, 3 equiv. of CB[7] and 3 equiv. of CB[8], complex conc 0.33 mM).

***Figure S31***. Corresponding zoom from data of Figure S28 for **VP-A-PV•**CB[7]_2_•CB[8]_2_ **_:_** e_CB7_ b_CB7_ f_CB7_ g_CB7_ d_CB7_ c_CB7_, and minor signals for **VP-A-PV•**CB[8]_4_ were observed and indicated as e_CB8_ b_CB8_ f_CB8_ g_CB8_ d_CB8_ c_CB8_.

Cross-peaks in negative phase (in blue) confirm the presence of dipolar couplings between the neighboring protons, which are indicated with dashed lines, and confirm the proton assignments. In addition, an exchange phenomenon between **VP-A-PV•**CB[7]_2_•CB[8]_2_ and **VP-A-PV•**CB[8]_4_ complexes led to cross-peaks in positive phase (in red) between the corresponding protons in the two complexes. The CB[7]/CB[8] exchange is observed on the aromatic proton signals where the CB inclusion highly affects the chemical shifts. Exchange cross-peaks can be observed via EXSY NMR experiments when the rate of exchange is between 10^-2^ and 10^2^ s^-1^.^[5]^ Moreover small cross-peaks in negative phase probably resulting from a mixture of dipolar coupling and CB exchange are observed (for instance signal d_CB[7]_ of **VP-A-PV•**CB[7]_2_•CB[8]_2_ with signal e_CB[7]_ resulting from a dipolar coupling and e_CB[8]_ resulting from both dipolar coupling and EXSY signal).

***Figure S32***. Zoom of the ROESY NMR spectrum (6.7-9.5 ppm region) of **VP-A-PV•**CB[7]_2_•CB[8]_2_ (500 MHz, mixing time **200 ms**, prepared with 1 equiv. of **VP-A-PV**, 3 equiv. of CB[7] and 3 equiv. of CB[8], complex conc 0.33 mM, NMR signals of **VP-A-PV•**CB[7]_2_•CB[8]_2_ **_:_** e_CB7_ b_CB7_ f_CB7_ g_CB7_ d_CB7_ c_CB7_, minor signals of **VP-A-PV•**CB[8]_4_ were observed and indicated as e_CB8_ b_CB8_ f_CB8_ g_CB8_ d_CB8_ c_CB8_).

The mixing time of the ROESY spectra affects the intensities of cross-peaks resulting from dipolar coupling or EXSY. Here, a mixing time of 200 ms led to an intensity decrease of dipolar coupling signals but an intensity increase of EXSY signals, confirming the two 2D-NMR occurrences.

***24/ ^1^H NMR spectra of VP-A-PV•CB[7]_2_•CB[8]_2_ with DMA as selective CB[8] competitor.***

A 0.25 mM solution of **VP-A-VP**•CB[7]_2_•CB[8]_2_ was prepared by addition of 27 µL of a **VP-A-VP** stock solution at 5 mM in D_2_O, 82 µL of a CB[7] stock solution at 5 mM and 450 µL of D_2_O onto 0.73 mg of CB[8]. The 400 MHz ^1^H NMR spectrum reveals the presence of small signals assigned to **VP-A-VP**•CB[8]_4_ (signals labeled with “*”, see top of Figure S31).

Then 58 µL of a 10 mM solution of 3,5-Dimethyl-1-adamantanamine hydrochloride (**DMA**) were added and the resulting solution was analyzed on a 400 MHz spectrometer (see bottom of Figure S31).

***
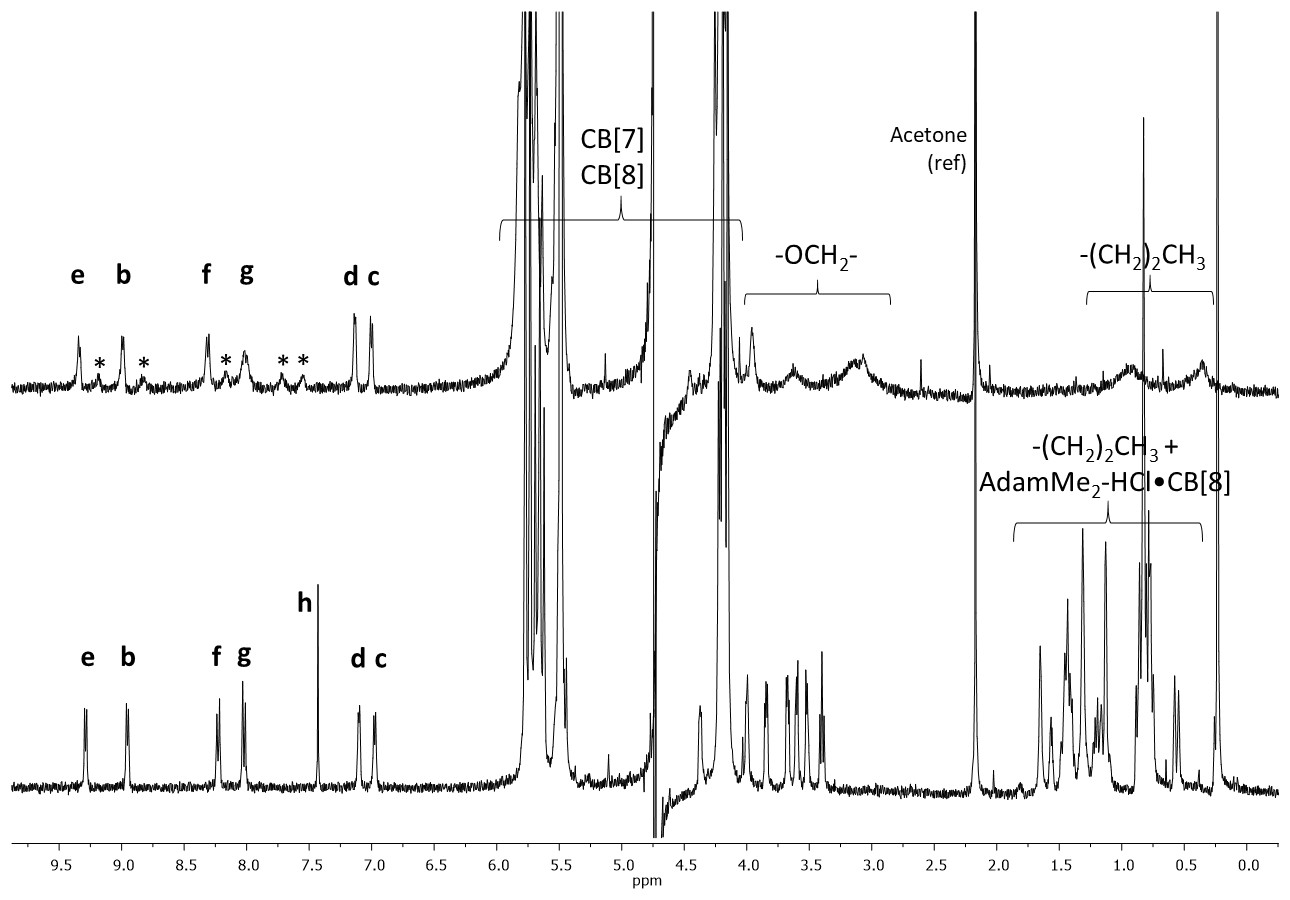
***

***Figure S33***. 400 MHz ^1^H NMR spectra of a solution of **VP-A-PV*•***CB[7]_2_•CB[8]_2_ before (top) and after (bottom) addition of **DMA** as a competitor. **DMA** captured the CB[8] macrocycles affording a new **VP-A-PV** complex, namely **VP-A-PV**•CB[7]_2_ with completely recovered flexibility of all guest fragments (signal of proton “h” totally recovered).

***25/ References.***

[1] D. Bardelang, K. A. Udachin, D. M. Leek, J. C. Margeson, G. Chan, C. I. Ratcliffe, J. A. Ripmeester, *Cryst. Growth Des.* **2011**, *11*, 5598–5614.

[2] H. Yin, R. Rosas, D. Gigmes, O. Ouari, R. Wang, A. Kermagoret, D. Bardelang, *Org. Lett.* **2018**, *20*, 3187-3191.

[3] H. Yin, R. Rosas, S. Viel, M. Giorgi, V. Monnier, L. Charles, D. Siri, D. Gigmes, Y. Nassar, F. Chevallier, C. Bucher, R. Wang, A. Kermagoret, D. Bardelang, *Angew. Chem. Int. Ed.* **2024**, *63*, e202315985.

[4] M. J. Abraham, T. Murtola, R. Schulz, S. Páll, J. C. Smith, B. Hess, E. Lindahl. *SoftwareX* **2015**, *1*, 19–25.

[5] a) Q. Cheng, H. Yin, R. Rosas, D. Gigmes, O. Ouari, R. Wang, A. Kermagoret, D. Bardelang, *Chem. Commun.* **2018**, *54*, 13825-13828; b) F. Liu, A. Kriat, R. Rosas, D. Bergé-Lefranc, D. Gigmes, S. Pascal, O. Siri, S. Liu, A. Kermagoret, D. Bardelang, *Org. Biomol. Chem.* **2023**, *21*, 9433-9442.

[6] Y. Ling, J. T. Mague, A. E. Kaifer, *Chem. Eur. J.* **2007**, *13*, 7908-7914.
